# Shape-defined poly(lactic acid)-nanohydroxyapatite composite microparticles modulate osteogenic differentiation in 3D microtissues of human mesenchymal stromal cells

**DOI:** 10.1016/j.mtbio.2026.103075

**Published:** 2026-03-26

**Authors:** Ke Song, Maryam Parvizifard, David Barata, Jiaping Li, Roman Truckenmüller, Pamela Habibović, Zeinab Niloofar Tahmasebi Birgani

**Affiliations:** MERLN Institute for Technology-Inspired Regenerative Medicine, Maastricht University, P.O. Box 616, Maastricht, 6200 MD, the Netherlands

**Keywords:** Composite biomaterials, Microparticle shape, Poly(lactic acid), Nanohydroxyapatite, Osteogenic differentiation, 3D microtissue

## Abstract

Microparticles have gained significant attention as promising injectable fillers for tissue defect repair, particularly in bone regeneration. While tailoring microparticle chemistry to guide osteogenic differentiation and bone regeneration has been extensively studied, the influence of microparticle shape remains less explored. We hypothesized that, similar to chemistry, microparticle shape can modulate the osteogenic differentiation of human mesenchymal stromal cells (hMSCs). To test this, we employed a micromolding method to fabricate shape-defined microparticles from poly(lactic acid) (PLA) or PLA-nanohydroxyapatite (nHA) composites with different aspect ratios and sizes, and then co-cultured them with hMSCs to self-assemble into 3D microtissues. Microtissues containing composite microparticles showed significantly higher alkaline phosphatase activity, with high-aspect-ratio and small-sized microparticles eliciting the strongest response. Both microparticle shape and composition regulated hMSC osteogenic differentiation according to gene expression analysis. In the absence of nHA, PLA microparticles with higher aspect ratios significantly increased the expression of osteogenesis-related genes, including *IBSP*, *SPP1*, and *MMP13*, whereas others showed minimal effects. Introducing nHA altered this trend, with small-sized microparticles inducing the highest *SPP1* expression and osteopontin production at late time points. Small-sized microparticles further promoted the expression of vinculin and yes-associated protein. Furthermore, etching composite microparticles to expose nHA on their surface amplified this size-dependent effect, leading to enhanced expression of the late osteogenic marker, *BGLAP,* in hMSC microtissues containing small cube composite microparticles. Our findings establish microparticle shape, especially size and aspect ratio, as fundamental design parameters that synergize with microparticle composition to direct hMSCs toward osteogenic lineage, offering a promising strategy for engineering injectable fillers for bone regeneration.

## Highlights

• Micromolding enabled fabrication of shape- and chemistry-tuned microparticles.
• Rectangular prism-shaped polymeric microparticles induced the highest osteogenic gene expression.
• Hydroxyapatite addition made small cubic microparticles most potent in osteogenesis.
• Microparticle shape and chemistry synergistically drive osteogenesis in 3D tissues.

## Graphical abstract

**Figure:**
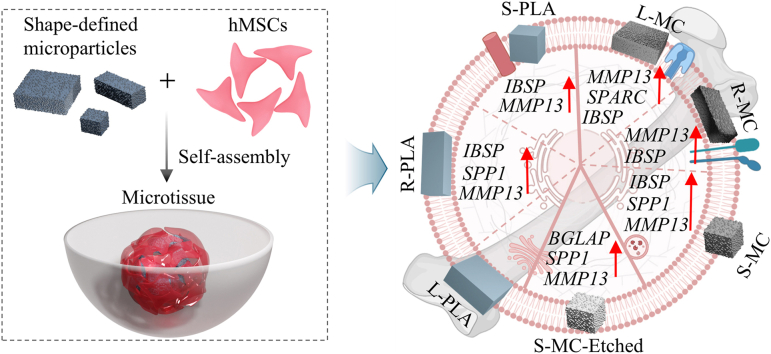

## 1 Introduction

Biomaterial microparticles have gained significant attention in different regenerative medicine applications, particularly for controlled release of growth factors [1,2], antibacterial agents [3], and anti-cancer drugs [4,5], or as microcarriers in cell expansion [6,7]. Another prominent application of microparticles is their use as bioactive fillers that can be inserted into defects to directly support tissue repair, particularly in bone regeneration and defect healing [8,9]. Microparticles offer a large surface area for interactions with cells and enable precise *in situ* injection and cell immobilization, making them an attractive biomaterial for defect filling and minimally invasive treatments. Moreover, their chemical composition and physical properties can be tailored to actively guide cell differentiation and the tissue regeneration process. For example, microparticles composed of bioactive materials, such as calcium phosphates (CaPs), have been shown to enhance the alkaline phosphatase (ALP) activity in human mesenchymal stromal cells (hMSCs), which are osteoblast precursors, thereby promoting their osteogenic differentiation *in vitro* [10,11]. Modifying the chemical composition of microparticles with bioactive factors, such as growth factors, exosomes, and peptides, has also proven effective in modulating bone regeneration processes, including immune response, angiogenesis, and osteogenic differentiation, both *in vitro* and *in vivo* [2,9].

Among various physicochemical characteristics of microparticles, their shape has emerged as a particularly intriguing property influencing their interactions with different cells [12], such as endothelial [13] and immune cells [14]. For example, improved *in vitro* sprouting of endothelial cells and *in vivo* vascular cell invasion in granular hydrogels was observed when they were developed with rod-shaped hydrogel microparticles rather than the spherical ones [13]. Previous studies have established that hMSCs and osteoblasts are also responsive to geometrical cues. For example, it has been shown that modifying the surface topography of flat substrates and microparticles can direct the osteogenic differentiation of hMSCs, and ultimately, promote osseointegration and bone-forming ability of the biomaterials, respectively [15,16]. Hence, we hypothesize that the three-dimensional (3D) geometry of microparticles can be similarly tuned to guide cell osteogenic differentiation and inform the design of more effective bone regenerative therapies. Specifically, the size and aspect ratio of microparticles are considered as key parameters in this study, for their potential to influence cell behavior. For example, the size of microparticles composed of polystyrene and biphasic calcium phosphate ceramic was reported to affect cell adhesion, spreading, proliferation, and even bone-forming ability [17,18]. Additionally, microstructures in high aspect ratios have been reported to guide cell alignment and cytoskeletal organization [19,20], which are critical regulators of osteogenic differentiation in hMSCs [21].

To explore this, we fabricated shape-defined microparticles from poly(lactic acid) (PLA), a biodegradable polyester widely used in bone tissue engineering [22,23], as well as PLA-CaP composites with different PLA/CaP ratios. CaPs, such as hydroxyapatite (HA), are primary candidates for developing synthetic bone graft substitutes due to their chemical similarity to the mineral phase of bone, and reported osteoconductive, and in some cases, osteoinductive properties, coupled with tunable degradation rates [24,25]. PLA-HA composites, utilized in various forms including porous scaffolds [26,27] and filler particles [28], demonstrate enhanced bioactivity, and compressive and tensile strength with reduced brittleness compared to pure HA, making them promising candidates for bone graft substitutes.

**Fig. 1:**
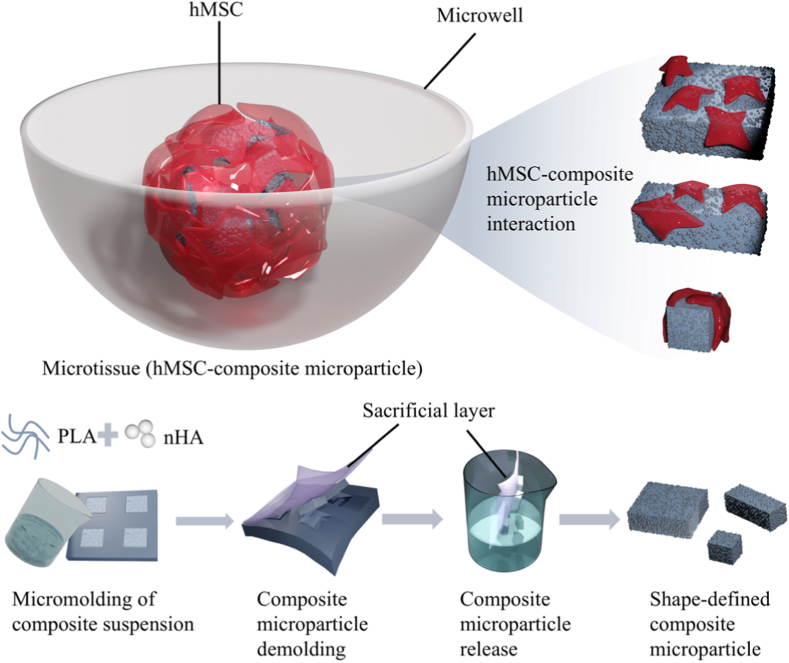
Schematic representation of engineering shape-defined PLA-nano HA (nHA) composite microparticles using a micromolding method, which were then used to generate hybrid microtissues composed of hMSCs and composite microparticles in non-adherent microwells.

Various fabrication methods, such as batch emulsion [29] and electrospraying [30], were previously employed to produce spherical PLA-HA composite microparticles, offering limited control over microparticle shape. Droplet microfluidics [31] has been applied to generate particles with certain shapes from low-viscosity fluids, with a limited range of geometries enabled. Furthermore, these methods are limited in generating microparticles from concentrated, high-viscosity (composite) slurries where viscous forces can prevent the spontaneous jet fragmentation and pinch-off required for uniform droplet formation. Shape-defined microparticles made of stiff polymers, such as PLA, were fabricated recently using a wettability contrast-based patterning platform [32], which only allowed for the production of ultrathin microparticles with a thickness of less than 0.5 μm. Another method established for fabricating shape-defined particles from different polymers is a lithographic micromolding method known as particle replication in non-wetting templates (PRINT), which employs geometrical, elastomeric molds with low surface energies to shape particles with defined geometries [33]. In this method, the particles are made by filling the molds with a pre-particle liquid precursor and its consequent solidification via photopolymerization or solvent evaporation, resulting in monodisperse particles with high fidelity to the mold geometry. The PRINT method was originally developed for the fabrication of shape-defined nanoparticles from different polymeric biomaterials, including poly(ethylene glycol), PLA, and poly(pyrrole) [[33], [34], [35], [36]]. Here, we adopted the PRINT method to fabricate PLA and PLA-HA microparticles with three distinct shapes. The shape-defined microparticles were co-aggregated with hMSCs in non-adherent microwells, facilitating the formation of hybrid 3D hMSC-microparticle microtissues (Fig. 1), as was reported by us earlier [11,37]. We then investigated the interactions between hMSCs and microparticles within the 3D environment, focusing on how microparticle shape influences *in vitro* osteogenic differentiation of hMSCs and whether these effects depend on the bioactivity of the microparticle chemistry.

## 2 Materials and methods

### 2.1 Fabrication of non-wetting micromolds

To develop the non-wetting micromolds, a master template was first fabricated using SU-8 photolithography method. Briefly, a photomask was designed in Clewin 4.0 and printed on a polymer film by Micro Lithography Services. Two-dimensional (2D) projections of three microparticle shapes, including large squares (100 μm × 100 μm), small squares (30 μm × 30 μm), and rectangles (30 μm × 100 μm), were included in the film photomask. These 2D patterns were later used to project UV through them and create 3D structures, thus transferring the patterns from the photomask to the SU-8 mold. In accordance with the data sheet provided by the SU-8 manufacturer, SU-8 50 (Kayaku Advanced Materials) was spin-coated onto silicon (Si) wafers (diameter: 4”, thickness: 525 μm, one side polished, Si-Mat Silicon Materials) at 3000 or 2500 rpm to obtain a film with a thickness of approximately 40 or 50 μm, respectively. The wafers were then pre-baked at 50, 65, and 95 °C for 10, 10, and 20 min, respectively, then cooled down to 25 °C at 2.5 °C/min, exposed to UV light at 365 nm for 10 s (UV-KUB 2, KLOE), and post-baked at 50, 65, and 80 °C for 5, 5, and 10 min, respectively. The SU-8 structures on the wafer were developed in propylene glycol monomethyl ether acetate (PGMEA, Sigma-Aldrich), rinsed with isopropyl alcohol, and blow-dried with nitrogen. The arrays of microparticle-shaped SU-8 features were then replicated onto intermediate poly(dimethyl siloxane) (PDMS) substrates. To fabricate the intermediate PDMS substrate, the SYLGARD^TM^ 184 silicone elastomer base (Dow) was mixed vigorously with its curing agent (Dow) with a 10:1 w/w ratio, poured onto the master mold, degassed in a vacuum chamber, cured at 70 °C for 2 h, and gently demolded to obtain a PDMS substrate with inverse patterns of the SU-8 structure. The PDMS substrate was then treated with oxygen plasma (FEMTO, Diener electronic) at 140 W for 5 min, followed by vapor deposition of 1H,1H,2H,2H-Perfluorooctyltriethoxysilane (FOTS, Sigma-Aldrich) under vacuum overnight. A second PDMS casting step was then performed as described previously to obtain the PDMS substrate equivalent to the SU-8 master with microparticle-shaped protrusions. A third perfluoropolyether urethane methacrylate (PFPE, Fluorolink MD700, Acota) substrate was replicated from this PDMS substrate, featuring the desired microparticle-shaped, non-wetting micromolds. The PFPE-based substrate was obtained by mixing PFPE solution and 1-hydroxycyclohexyl phenyl ketone (Sigma-Aldrich) with a 100:1 v/w ratio, pouring the mixture onto the PDMS intermediate substrate, crosslinking it in a UV chamber (UVPTM CL-1000 Ultraviolet Crosslinker, Fisher Scientific) at 1.2 mJ/cm^2^ under inert nitrogen atmosphere for 30 min, and finally, gently demolding the cured substrate.

### 2.2 Fabrication of PLA-nHA composite microparticles

Various amounts of PLA (PURASORB PDL, IV(dl/g):0.5, Corbion) (10%, 30%, 50% or 70% w/v) and nano-sized HA powder (nHA, Kuros Biosciences) [38] (10%, 30%, 50% or 70% w/v) were dissolved and suspended in acetonitrile (TCI), respectively, to reach the composite slurries with PLA/nHA ratios of 70/10, 50/30, 30/50, 10/70 w/w. 70% w/v PLA solution was used as a control. Composite suspensions were magnetically stirred for 24 h before use. To fabricate PLA-nHA composite microparticles, a poly(ethylene terephthalate) (PET) film (thickness: 0.2 mm, Sigma-Aldrich) was used to cast approximately 20 μL of composite suspensions into the micromolds formed on the PFPE substrates (2 cm × 4 cm). This was followed by acetonitrile evaporation and consequent solidification of the composite microparticles inside the micromolds. To demold the composite microparticles, a 4% w/v poly(vinyl alcohol) (PVA, Mw∼2000, TCI) solution was poured onto the PFPE substrate in order to create a sacrificial layer, which was subsequently dried overnight, peeled off, thereby removing the composite microparticles, and dissolved in water to release free-standing composite microparticles (Fig. 1). Composite microparticles were collected by filtering through stacked cell strainers with 150- (pluriSelect) and 40 μm-pores (Corning). PLA microparticles were fabricated using the same method. Surface-etched composite microparticles were made by immersing the composite microparticles with a PLA/nHA of 30/50 w/w in a 0.25 M sodium hydroxide (NaOH)/ethanol (30/70 v/v) solution for 3 min, washed three times in deionized (DI) water and air dried.

### 2.3 Physicochemical characterization of composite microparticles

#### 2.3.1 Wettability

The wettability of composites was measured by the sessile drop technique with an optical contact angle device (OCA15, Data-physics). Briefly, composite films with the same compositions described in section 2.2 were formed. In this experiment, we used composite films instead of composite microparticles, since the latter did not provide sufficient area for setting the water drops. A sessile Milli-Q water drop was deposited onto each film's surface with a syringe, and the drop contour was fitted by the Young-Laplace method. The dynamic measurement was recorded, and the contact angles of the composites were quantified after 0, 10, 20, and 40 s. Three independent contact angle measurements were performed for each sample.

#### 2.3.2 Morphology

A scanning electron microscope (SEM, Jeol JSM-IT200, JEOL) was used to observe the morphology of the composite microparticle. The composite microparticles were mounted onto standard pin aluminum stubs using conductive carbon adhesive tabs (Electron Microscopy Sciences). After sputtering the samples with a gold coating layer using a high vacuum sputter coater (SC7620, Quorum Technologies), composite microparticles were observed with SEM at a working distance of 11 mm, under an accelerating voltage of either 10 or 20 kV.

#### 2.3.3 Chemical composition

Attenuated total reflection Fourier transform infrared spectroscopy (ATR-FTIR, Nicolet iS50, Thermo Fisher Scientific) was employed to determine the chemical bonds present in composite microparticles within the wavenumber region of 400 to 3500 cm^−1^, with a step size of 0.5 cm^−1^. Energy dispersive X-ray spectroscopy (EDS, coupled with SEM) was used to measure the elemental composition and distribution in the microparticles. For EDS, all samples were prepared as described for SEM analysis and analyzed at an accelerating voltage of 20 kV. The atomic contents of carbon, oxygen, calcium, and phosphorus were quantified in the composite microparticles. Additionally, the distributions of these elements were mapped in the composite microparticles with a PLA/nHA ratio of 30/50 w/w. All measurements were repeated independently at least three times.

#### 2.3.4 Surface profile and roughness

The composite microparticles and their PFPE molds were scanned with a confocal 3D laser scanning profilometer microscope (VK-X200, Keyence) integrated with MultifileAnalyzer software (Keyence) for image analysis. Height maps of the surface of the composite microparticles were obtained and the average height of the composite microparticles was measured in 18 microparticles from three independent replicates. In addition, for each condition, the arithmetic mean height of surface (Sa), an indicator of surface roughness, was calculated in 15 microparticles from three independent replicates.

#### 2.3.5 In vitro degradation

1 mg of composite microparticles was placed in cell strainers and incubated in 9 mL of Minimum Essential Medium α (α-MEM, no nucleosides, Gibco), supplemented with 10% v/v fetal bovine serum (FBS, Gibco), at 37 °C and 5% CO_2_. On days 15 and 30 after incubation, composite microparticles were collected and washed with deionized water three times. After air-drying, the morphology of the composite microparticles was observed using SEM as described above.

### 2.4 Formation and characterization of hybrid hMSC-composite microparticle microtissues

#### 2.4.1 Microwell fabrication and characterization

3D microtissues were formed in non-adherent U-shaped microwells, which were fabricated using a free-forming variant of the negative pressure thermoforming method [39,40]. Briefly, brass molds containing arrays of cylindrical holes with diameters of 550 μm were first fabricated using micromilling. Then, in-house-made, transparent polycarbonate (PC) films with 50-μm thickness were stretched into the holes of the micromilled brass mold at a temperature of 153 °C and negative pressure of 15 bar. After 1 min, the pressure was released and the thermoformed films were demolded. The generated PC microwell arrays were punched to the size fitting either 96- or 24-well cell culture plates. The thermoformed PC microwell arrays were inspected using the 3D confocal laser scanning profilometer microscope and SEM, as described above.

#### 2.4.2 Cell culture

Bone marrow-derived hMSCs were isolated from a single donor who had given consent, as described earlier [41]. The use of the hMSCs in the culture experiments of this study complied with the relevant laws and institutional guidelines and was based on a corresponding (signed) document from the Medical Ethical Committee (Medisch Ethische Toetsingscommissie) of the Medisch Spectrum Twente hospital, Enschede, The Netherlands. hMSCs were cultivated in a tissue culture flask at a density of 1000 cells/cm^2^ in a basic medium (BM) at 37 °C and 5% CO_2_. The BM was composed of α-MEM supplemented with 10% v/v FBS (lot BCBT6987, Gibco) and 0.2 mM L-ascorbic acid 2-phosphate magnesium salt (Sigma-Aldrich). The cell culture medium was refreshed every two days. Once the cells reached 70%-80% confluency, they were trypsinized and used for the formation of microtissues.

#### 2.4.3 Formation of microtissues

Prior to cell seeding, microwell arrays thermoformed on PC films were sterilized three times by rinsing with 70% ethanol, washed with phosphate buffer saline (PBS), placed in 96- or 24-well plates, and fixed in their place using O-rings (ERIKS). Subsequently, a 1% w/v Pluronic F108 (Sigma-Aldrich) solution was added to each well and incubated at 37 °C overnight, in order to obtain microwells with non-adherent surfaces, preventing the adhesion of cells and proteins [11]. After removing the Pluronic F108 and several washing steps in PBS, the microwell arrays were ready for cell seeding. hMSCs (passage 4-6) and composite microparticles were co-seeded at densities of 2000 cells and 10 microparticles per microwell for large cube microparticles. For other microparticle shapes, the number of microparticles seeded per microwell was adjusted to maintain an equivalent total microparticle volume in each microtissue. Either BM or osteogenic medium (OM, *i.e.*, BM supplemented with 10 nM dexamethasone (Sigma-Aldrich)), both supplemented with 100 U/mL penicillin and 0.1 mg/mL streptomycin, was added to the cultures. The cell culture medium was refreshed every two days. Cell-only microtissues served as controls in all experiments. The microtissue formation and morphology of the hybrid microtissues were monitored under an optical microscope (CKX53, Olympus) with bright-field images taken on days 1 and 10. The geometrical properties of microtissues, including Feret's diameter and roundness, were quantified using Fiji 1.54f analyzer (https://fiji.sc/). For each condition, 30 spheroids from three biological replicates were imaged and quantified.

#### 2.4.4 Metabolic activity and viability of hMSCs in microtissues

The metabolic activity of hMSCs in microtissues in both BM and OM was measured using PrestoBlue (Thermo Fisher Scientific) assay on days 3, 7, and 10. According to the manufacturer's instructions, cell culture medium was removed; 200 μL of PrestoBlue reagent diluted in cell culture medium (1/10 v/v) was added to the microtissues in each well, and incubated for 30 min at 37 °C and 5% CO_2_. Then, the supernatant was collected and its fluorescence intensity was measured using a microplate reader (CLARIOstar, BMG LABTECH) at excitation and emission wavelengths of 560 and 590 nm, respectively. The viability of hMSCs in hybrid microtissues was assessed on day 10 in both BM and OM using LIVE/DEAD Fixable Dead Cell Stain Sampler Kit (Thermo Fisher Scientific) according to the manufacturer's instructions. Hybrid microtissues were first fixated in formaldehyde (FA, 4%, v/v) solution for 30 min, rinsed with PBS three times, permeabilized with 0.1% (w/v) Triton-X 100 (Sigma-Aldrich) for 30 min, and blocked with 2% (w/v) bovine serum albumin (BSA, Sigma-Aldrich) for 1 h. Then, the microtissues were incubated with 33 nM phalloidin (Alexa Fluor 647, Thermo Fisher Scientific) in 0.1% BSA at 4 °C overnight, and 7 μg/mL 4′,6-diamidino-2-phenylindole (DAPI, Sigma-Aldrich) at room temperature for 45 min to label cytoskeletal F-actin and cell nuclei, respectively. Images were acquired using a confocal laser scanning fluorescence microscope (TCS SP8 STED, Leica Microsystems) as scanning stacks with 2 μm-thick slices.

#### 2.4.5 ALP activity of hMSCs in microtissues

The ALP activity of hybrid microtissues cultured in either BM or OM for 10 days was assessed using the ALP Fluorescent Assay Kit (Thermo Fisher Scientific) according to the instructions of the manufacturer. Briefly, 100 μL of 4-methylumbelliferyl phosphate disodium salt reagent was added to the hybrid microtissues in each well and incubated for 30 min at room temperature. The supernatant was then collected, and its fluorescence intensity was determined using the CLARIOstar plate reader at excitation and emission wavelengths of 386 and 448 nm, respectively. Microtissues were further lysed in proteinase K (Sigma-Aldrich) overnight at 56 °C, and the DNA content of the lysate was determined using the CyQUANT Cell Proliferation kit, according to the data sheet of the manufacturer. The ALP activity of each sample was normalized to the corresponding DNA content.

#### 2.4.6 Immunocytochemical analysis of osteogenic proteins in microtissues

Microtissues without or with microparticles were cultivated in OM and harvested on day 10. After washing with PBS, the microtissues were fixated with 4% v/v FA for 20 min, permeabilized with 0.3% v/v Triton X-100 for 30 min, and their non-specific binding sites were blocked with a blocking solution (5% v/v goat serum and 3% v/v bovine serum albumin in PBS) for 1 h. Samples were incubated with unconjugated rabbit-anti-human recombinant ALP polyclonal primary antibody (1:300, Thermo Fisher Scientific) and unconjugated mouse-anti-human osteopontin (OPN) monoclonal primary antibody (1:200, Thermo Fisher Scientific) at 4 °C overnight. Subsequently, samples were rinsed with PBS to remove unconjugated antibodies, followed by overnight incubation with goat anti-rabbit secondary antibody (Alexa Fluor 488, 1:500) for ALP visualization, goat anti-mouse secondary antibody (Alexa Fluor 568, 1:500) for OPN visualization, and phalloidin (Alexa Fluor 647, 33 nM) for cytoskeletal F-actin labeling. Finally, the samples were counterstained with 4',6-diamidino-2-phenylindole (DAPI, 7 μg/mL) and imaged using fluorescence confocal microscopy as scanning stacks with 2 μm-thick slices. ALP and OPN fluorescence intensities, and the number of nuclei per microtissue were quantified on 3D-projected confocal images using Fiji software.

#### 2.4.7 Morphology of microtissues

Hybrid microtissues were harvested on day 10, washed with PBS, fixated with 4% v/v FA solution, dehydrated in ethanol solutions with gradient concentrations (30%, 50%, 70%, 80%, 80%, 90%, and 95% v/v) for 10 min each, and in 100% ethanol overnight, incubated in hexamethyldisilane (HMDS, Sigma-Aldrich) for 30 min and dried at room temperature. The microtissues were then mounted onto an aluminum stub, sputter-coated with gold and inspected using SEM, as described earlier.

#### 2.4.8 mRNA expression of osteogenic genes in microtissues

The mRNA expressions of a panel of osteogenesis-related genes, including *SPP1*, *MMP13*, *ALPL*, *COL1A1*, *BMP2*, *RUNX2*, *BGLAP*, *TGFB1*, *MGP*, *SP7*, *PDPN*, *IBSP*, and *SPARC* (full names introduced in Table S1), in hMSCs in microtissues were evaluated by quantitative reverse transcription polymerase chain reaction (RT-qPCR). Microtissues were collected on days 5, 10, and 20 for RNA isolation using Trizol reagent combined with PureLink RNA Mini Kit (Thermo Fisher Scientific). Then, the isolated RNA was used to amplify 20 ng of cDNA using iQ SYBR Green Supermix (Bio-Rad) in a CFX96 Real-Time PCR Detection Kit (Bio-Rad). Quantification of transcription levels was performed through ΔΔCt method using *TBP* as the housekeeping gene. Primer sequences of the selected genes and their annealing temperatures are listed in Table S1. The mRNA fold expressions were calculated by normalization to cell-only microtissues (control) on the same day. Cluster heatmaps and principal component analysis (PCA) graphs were plotted using the SRplot platform [42].

#### 2.4.9 Quantification of protein expression in microtissues

The microtissues were collected on days 5, 10, and 20 for protein extraction and analysis. At each time point, the microtissues were lysed with ProcartaPlex™ Cell Lysis Buffer (Thermo Fisher Scientific), centrifuged at 14000 rpm for 10 min, and the resulting supernatant was collected. The expression of receptor activator of nuclear factor-κB ligand (RANKL), OPN, growth arrest-specific 6 (GAS6) and matrix metalloproteinase 13 (MMP13) were detected using xMAP bead-based technology (ProcartaPlex custom panel, Invitrogen, Thermo Fisher Scientific). Protein concentrations were measured using a Luminex100 (Bio-Rad, Veenendaal) with data acquisition in Bio-Plex Manager 6.0 software (Bio-Rad, Veenendaal), as reported previously [43].

#### 2.4.10 Assessment of hMSC mechanotransduction in 2.5D cell culture

To elucidate how microparticle shapes, specifically size and aspect ratio, influence hMSC mechanotransduction and physical adaptation, a two and a half-dimensional (2.5D) culture was utilized. In detail, hMSCs were seeded at a density of 10000 cells per well in 24-well plates containing glass coverslips, onto which microparticles from different groups had been deposited. Cells were cultured in OM for 10 days. Cells cultured on glass coverslips without microparticles served as controls. On day 10, samples were fixed with 4% v/v paraformaldehyde for 10–15 min at room temperature, permeabilized with 0.1% v/v Triton X-100 in PBS, and blocked with 5% v/v normal donkey serum in PBS. Samples were then incubated overnight at 4 °C with primary antibodies against vinculin (VCL, Thermo Fisher Scientific) and yes-associated protein (YAP, Proteintech). After washing, samples were incubated with species-appropriate fluorescent secondary antibodies. F-actin was stained with fluorophore-conjugated phalloidin, and nuclei were counterstained with DAPI. Z-stack images were acquired with identical acquisition settings applied across all groups, with selected stacks to focus on cells that attached to the particles. Quantitative analysis of VCL and YAP fluorescence intensities, as well as nuclear and cytoskeletal morphological parameters, was performed using CellProfiler software (version 4.2.1) on selected sections of Z-stack images containing cells directly attached to microparticles.

### 2.5 Statistical analysis

The quantitative data was presented as the mean ± standard deviation. All biological experiments were performed with 3 independent samples unless indicated otherwise. GraphPad Prism 10.0 software was used for statistical analysis. To that end, the data were analyzed by one- or two-way analysis of variance (ANOVA) followed by Tukey's honestly significant difference (HSD) post-hoc test for multiple comparisons. The differences between the results were considered statistically significant when the p-value was less than 0.05, and asterisk signs ‘∗’, ‘∗∗∗’, ‘∗∗∗’, and ‘∗∗∗∗’ represented *p* < 0.05, *p* < 0.01, *p* < 0.001, and *p* < 0.0001, respectively.

## 3 Results and discussion

### 3.1 Fabrication and characterization of composite microparticles

**Fig. 2:**
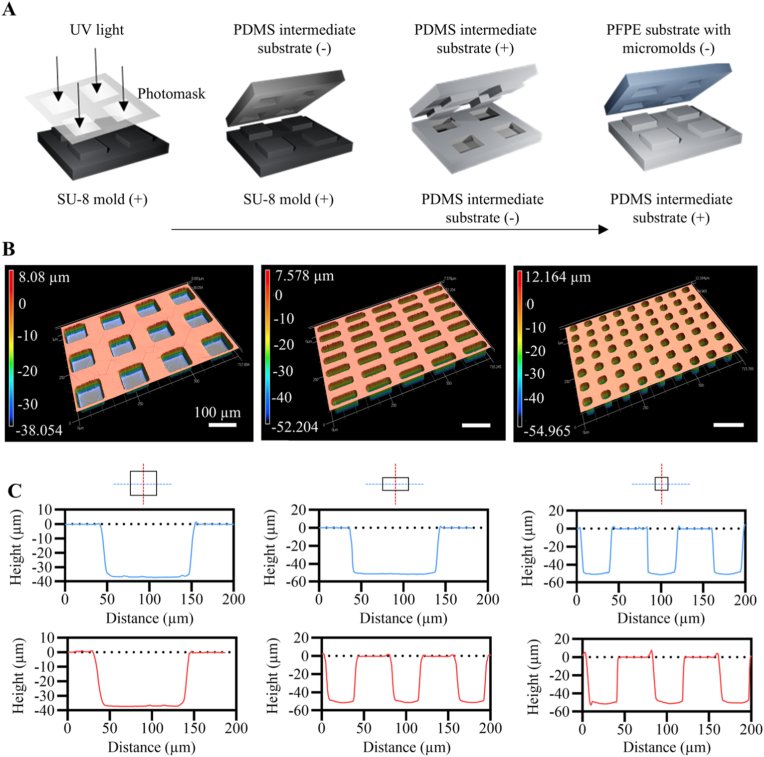
Fabrication of non-wetting PFPE micromolds used for composite microparticle replication. (A) Schematic representation of fabrication process of the PFPE substrate containing micromolds using SU-8 photolithography, followed by a two-step PDMS replica molding and a PFPE replica molding step. In each step, “+” and “–” signs indicate whether the produced microparticle-shaped structures protruded from or were indented into the corresponding substrate, respectively. (B) Height maps and (C) line profiles of the PFPE substrates containing various micromolds obtained by confocal laser scanning profilometer microscope. Indications above the scale bar apply to all images in the same row.

To engineer shape-defined composite microparticles, patterned substrates containing micromolds with different geometries were first fabricated using PFPE. PFPE-based substrates were previously employed in precision fabrication methods, such as soft lithography [44], due to their solvent resistance, chemical stability, and higher Young's modulus compared to typical PDMS-based stamps used in these methods. More importantly, the PFPE-based substrates show an extremely low surface energy (12-20 mN·m^−1^) [44,45], enabling easy detachment from other materials during demolding steps [46,47]. In our study, low-surface energy PFPE substrates containing the micromolds were successfully fabricated by replicating PDMS intermediate substrates that were in turn fabricated by replica molding from an SU-8 master (Fig. 2A) [48,49]. This approach enabled multiple replications of the final PFPE substrates containing micromolds. The micromolds had the dimensions of 100 μm × 100 μm × 40 μm (large cube, L), 30 μm × 30 μm × 50 μm (small cube, S), and 30 μm × 100 μm × 50 μm (rectangular prism, R) (Fig. 2B and C), which subsequently were used to produce composite microparticles with the same shapes, labeled as L-MC, S-MC and R-MC, respectively (MC indicating microparticle-composite). To generate the composite microparticle, homogenous PLA-nHA suspensions at different PLA/nHA (70/0 (or PLA), 70/10, 50/30, 30/50, and 10/70, w/w) were used. We first created films made of composite materials and analyzed their wettability using contact angle measurement. In all groups, the water contact angle decreased over time, an effect that was accentuated in conditions with high nHA content (Fig. S1A, B). This reflects hydrophilic contribution by HA's hydroxyl groups [50,51] as shown before in PLA-HA composite scaffolds [52]. Here, we observed that 40 s after starting the measurements, the water contact angle of composite films with PLA/HA of 30/50 (49.61° ± 11.37°) and 10/70 (18.65° ± 14.58°) was lower than that of pure PLA (63.40° ± 6.0°), indicating enhanced wettability of the composites at high nHA contents. At lower nHA contents, the water contact angle on the composite film was similar or slightly higher than that on PLA film (77.78° ± 3.27° and 64.07° ± 14.26° for PLA/nHA of 70/10 and 50/30, respectively) (Fig. S1B). Interestingly, the PLA/nHA 10/70 composite demonstrated the highest initial water contact angle (106.15° ± 6.62° at 0 s), which may be due to its surface roughness [53] formed as a consequence of nHA addition. In summary, compared to pure PLA, composites with PLA/nHA of 30/50 and 10/70 demonstrated higher wettability, indicating their potential for enhanced cell adhesion [54].

**Fig. 3:**
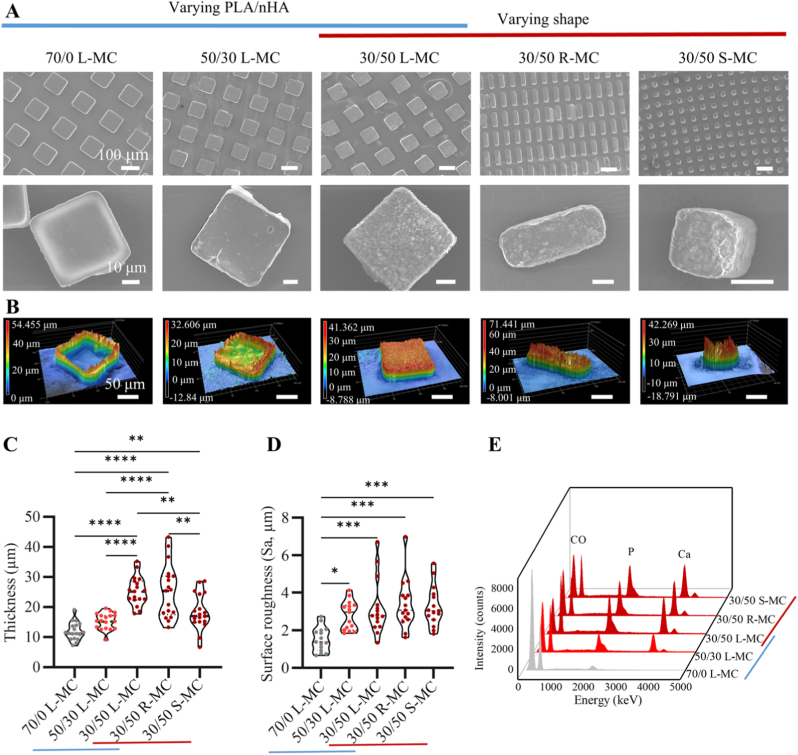
Characterization of shape-defined microparticles with different PLA/nHA ratios and shapes. (A) SEM images of the PLA microparticles (70/0 L-MC), and 50/30 L-MC, 30/50 L-MC, 30/50 R-MC, and 30/50 S-MC composite microparticles. Indications above the scale bars apply to the images in the same row. (B) Height maps of PLA and composite microparticles with different PLA/nHA and shapes. Each map corresponds to the microparticle group that is presented above it. Indications above the scale bar apply to all images. (C) Average thickness (n = 15), (D) surface roughness (n = 18), and (E) EDS spectra of PLA and composite microparticles with different PLA/nHA and shapes. In EDS spectra, C, O, P and Ca indicate one of the main peaks corresponding to carbon, oxygen, phosphorus, and calcium elements, respectively. Data in C and D were analyzed using a one-way ANOVA followed by Tukey's HSD post-hoc test (∗*p* < 0.05, ∗∗*p* < 0.01, ∗∗∗p < 0.001, and ∗∗∗∗*p* < 0.0001). For all microparticles the labels indicate the PLA/nHA ratio and shape.

The PLA-nHA slurries were cast onto the micromolds by compression with a PET sheet that has a higher surface energy (≈40 mN·m^−1^) [55] compared to PFPE, and therefore, could effectively push the suspension into the micromolds and remove any residual material. This eventually led to the formation of discrete microparticles with the various PLA/nHA ratios inside the micromolds upon solvent evaporation and suspension solidification. These could be demolded from the PFPE substrates as arrays of shape-defined microparticles (Fig. 3A, top row, for all composite microparticles the labels indicate the PLA/nHA ratio and shape). In addition, we fabricated composite microparticles with three distinct geometries described earlier with a PLA/nHA of 30/50 ratio. In all conditions, the composite microparticles accurately replicated the geometries of the micromolds and could be released in water by sacrificing the PVA film used for their demolding (Fig. 3A, bottom row, Fig. S1C). The use of PRINT method has enabled the precision fabrication of shape- and size-uniform nano- or microparticles across a wide range of geometries and biomaterials [56], including poly(lactic-co-glycolic acid) [34] and different hydrogels [57]. In this study, we, for the first time, expanded the capacity of the PRINT method to bioactive composite biomaterials, specifically PLA-nHA composites with tunable nHA content. Notably, we succeeded in processing composite formulations with high nHA loading content (up to 70% w/v), a formulation that is highly challenging to handle due to unfavorable rheological properties and rapid drying. High HA content in PLA-HA composites is considered favorable for enhancing bioactivity and osteogenic properties, and promoting effective bone regeneration [58].

The surface profile of the microparticles was inspected using laser profilometry, revealing a pronounced meniscus in the center of pure PLA microparticles (Fig. 3B, Fig. S1D), which was likely generated due to the “coffee-ring” effect. This phenomenon is characterized by faster evaporation of the edges in a liquid drop compared to the central region, resulting in outward capillary flow of the liquid, from the center toward the edges, during the drying process [59]. When the nHA content reached 50% w/w, the “coffee-ring” effect was eliminated, suggesting the increased solid content compensating for volume loss during evaporation in the central region of the composite microparticles. The measurements of average thickness of the microparticles were also in line with these observations (Fig. 3C), showing that PLA/nHA 10/70 composite microparticles exhibited the highest average thickness (26.99 ± 3.72 μm), followed by PLA/nHA 30/50 composite microparticles (25.27 ± 4.71 μm), both significantly higher than those in PLA/nHA 50/30 composite microparticles (15.24 ± 2.73 μm) and pure PLA microparticles (11.85 ± 2.97 μm). Additionally, the results indicated no significant difference in the average thickness of PLA/nHA 30/50 composite microparticles with L-MC (25.27 ± 4.71 μm) and R-MC shapes (25.20 ± 8.88 μm). PLA/nHA 30/50 composite microparticles with S-MC shape had a significantly lower thickness of 18.25 ± 5.94 μm, compared to those with other shapes. This reduction may be attributed to limitations in air displacement in the confined space of the smallest micromolds during the casting process.

Surface roughness has been suggested to play a pivotal role in modulating hMSC osteogenic differentiation. For example, Faia-Torres et al. reported that hMSCs cultured on a polycaprolactone substrate with a surface roughness gradient, ranging from 0.5 to 4.7 μm, produced different amounts of osteogenic proteins, including ALP and type I collagen, across the substrate, with the regions with a roughness of 0.9 – 3.5 μm showing higher osteogenic protein production [60]. In the current study, the average Sa of 70/0 L-MC, 50/30 L-MC, and 30/50 L-MC, 30/50 R-MC and 30/50 S-MC was 1.52 ± 0.65 μm, 2.80 ± 0.76 μm, 3.19 ± 1.47 μm, 3.40 ± 1.32 μm, and 3.28 ± 1.08 μm, respectively (Fig. 3D), which fall within the range shown by Faia-Torres et al. to be suitable for inducing hMSC osteogenic differentiation. Naturally, increasing nHA content in the composite formulation increased the surface roughness of the microparticles.

Elemental composition of the composite microparticles, investigated using EDS, showed a positive correlation between nHA content and intensity of the main peaks of calcium and phosphorus (Fig. 3E). Furthermore, homogeneous element distributions were evident across the microparticle surfaces (Fig. S1E). FTIR spectra of the PLA/nHA 30/50 microparticles illustrated the absorption peaks at 564, 604, and 1034 cm^−1^, representative of the stretching bands of phosphate groups, confirming the presence of nHA in the composite microparticles (Fig. S2). Moreover, the stability of composite microparticles in cell culture medium for up to 30 days was inspected by imaging. While all composite microparticles retained their shapes, submicron and micron pores were seen in PLA, PLA/nHA 70/10, and PLA/nHA 50/30 groups, plausibly indicating the bulk degradation of PLA (Fig. S3) [61]. However, the composite microparticles with nHA contents of 50% and higher did not show this porous structure, indicating their higher stability under physiological conditions up to 30 days compared to their counterparts with no or lower nHA contents. Before being seeded with cells, the composite microparticles were sterilized with UV, which did not significantly alter their surface roughness (Fig. S4). In summary, we successfully fabricated shape-defined composite microparticles with varying PLA/nHA, which showed improved shape fidelity and higher stability in physiological conditions at higher nHA contents.

### 3.2 Formation and characterization of hMSC microtissues containing composite microparticles with different PLA/nHA

**Fig. 4:**
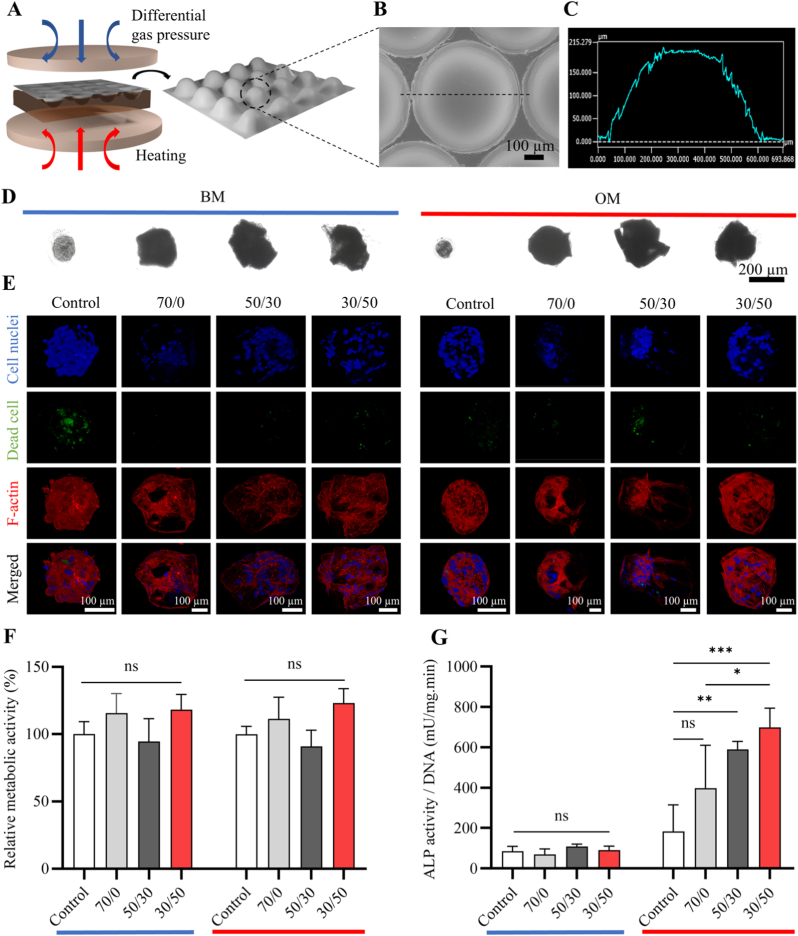
Formation and characterization of hybrid hMSC-composite microparticle microtissues. (A) Schematic representation of microwell array fabrication by microthermoforming of PC films. (B) SEM image of a microwell observed from the back side and (C) its height profile across a line drawn through the center of the microwell measured by a 3D laser scanning profilometer microscope. (D) Bright-field images of hybrid microtissues in BM and OM on day 10, indicated by blue and red lines above them, respectively. The images correspond to the microtissues presented below them. (E) Maximum intensity projections of confocal microscope images of hybrid microtissues in BM (left panel) and OM (right panel) on day 10. Cytoskeletal F-actin staining, cell nuclei and dead cells are visualized in red, green and blue, respectively. Scale bars apply to the images in the same column. (F) Metabolic activity relative to control and (G) ALP activity normalized to DNA content of hMSCs in hybrid microtissues in BM (indicated by blue line) and OM (indicated by red line). Data in F and G were analyzed using a one-way ANOVA followed by Tukey's HSD post-hoc test (∗*p* < 0.05, ∗∗*p* < 0.01, and ∗∗∗*p* < 0.001). ‘ns’ represents non-significant difference. (For interpretation of the references to colour in this figure legend, the reader is referred to the Web version of this article.)

To form hMSC-composite microparticle microtissues, arrayed microwells were thermoformed onto PC films (Fig. 4A). The microwells exhibited a hemispherical shape with a depth of ∼200 μm (Fig. 4B and C). After being rendered non-adherent for cells by Pluronic coating, the microwells were seeded with cells and composite microparticles, permitting the formation of spheroidal microtissues without (control) or with composite microparticles with L-MC shape and varying PLA/nHA, which were then maintained for up to 10 days (Fig. 4D, Fig. S5). Cell viability results revealed the existence of dead cells predominantly in the core of hMSC-only control microtissues, while all microtissues that contained microparticles showed higher cell viability (Fig. 4E). This improvement is likely attributed to the reduced cell density within microparticle-containing microtissues, which effectively mitigates the formation of a necrotic core. Notable exceptions were observed in microtissues containing PLA/nHA (50/30) in BM, where small regions of cell death were still detectable. We hypothesize that this formulation corresponds to a fast degradation rate compared to other groups (Fig. S3), therefore facilitating autocatalytic hydrolysis of PLA and transiently elevating local concentrations of acidic degradation products [62].

Compared to control, the organization of actin fiber was more visible in microtissue containing microparticles, which resulted from the mechanotransductive nature of biomaterials. Metabolic activity of hMSCs in microtissues was not affected by the introduction of composite microparticles (Fig. 4F). ALP activity, widely used as an early indicator of osteogenic differentiation [63,64], was generally higher in microtissues in OM than those in BM (Fig. 4G). More importantly, in the OM condition, incorporation of composite microparticles, specifically those with high nHA content, in the microtissues increased their ALP activity compared to control microtissues as well as microtissues containing pure PLA. Notably, in the OM condition, microtissues containing PLA/nHA 30/50 demonstrated the highest ALP expression among all groups, which was 3.82-fold and 1.76-fold higher than those in control microtissues and microtissues containing pure PLA, respectively (Fig. 4G), highlighting the important effect of nHA content on inducing osteogenic commitment in hMSCs. In summary, composite microparticles with varying PLA/nHA ratios were able to assemble with hMSCs spontaneously, forming viable 3D microtissues, and high nHA contents in composite microparticles were more favorable to increase hMSC ALP activity.

### 3.3 Formation and characterization of hMSC microtissues containing composite microparticles with different shapes

**Fig. 5:**
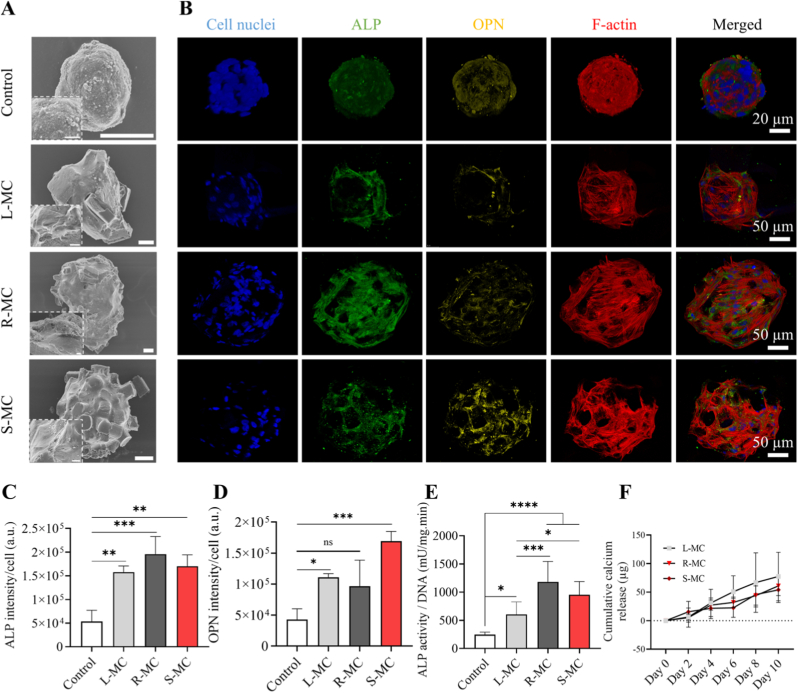
Formation and characterization of hMSC microtissues containing composite microparticles with PLA/nHA of 30/50 and different shapes. (A) SEM images of hMSC-composite microparticle microtissues in OM on day 10, indicating the morphology of the microtissues (main panels) and close-up of the cells at the surface of the microtissues (insets). Scale bars indicate 100 μm in the main panels and 10 μm in the insets. (B) Maximum intensity projections of confocal microscope images of hybrid microtissues in OM on day 10. Cell nuclei, ALP, OPN, and cytoskeletal F-actin are visualized in blue, green, yellow, and red, respectively. Scale bars apply to the images in the same row. Quantification of (C) ALP and (D) OPN fluorescence intensities per cell number, derived from maximum intensity projections of confocal images, and (E) ALP activity normalized to DNA content in hMSC microtissues. (F) Cumulative calcium (Ca^2+^) release from composite microparticles in cell culture medium. Data in C, D, and E were analyzed using a one-way ANOVA followed by Tukey's HSD post-hoc test (∗*p* < 0.05, ∗∗*p* < 0.01, ∗∗∗*p* < 0.001 and ∗∗∗∗*p* < 0.0001). ‘ns’ represents non-significant difference. (For interpretation of the references to colour in this figure legend, the reader is referred to the Web version of this article.)

Based on the results so far, microtissues containing hMSCs and PLA-nHA microparticles with a ratio of 30/50 cultured in OM were selected to investigate the effect of composite microparticle shape on hMSC osteogenic differentiation. PLA/nHA 30/50 composite microparticles with different shapes (L-MC, R-MC, and S-MC) were co-seeded with hMSCs in microwells, forming hybrid microtissues. These microtissues exhibited significantly larger Feret's diameters and lower roundness on both day 5 and day 10 compared to the cell-only control microtissues, with no significant differences observed among the composite microparticle-containing groups (Fig. S6A-B). The microtissue solidity, defined as the area of a microtissue divided by the area of its convex hull, appears to be dependent on microparticle shape on day 5, as microtissues containing L-MC exhibit significantly higher solidity than those containing R-MC and S-MC (Fig. S6C-D). This indicates that R-MC and S-MC led to more undulations in the outer shape of the microtissues. On day 10, however, no significant differences were seen among the solidity of various groups, likely due to microtissue compaction and cells completely engulfing the microparticles. Assessing the microtissue morphology with SEM on day 10 indeed showed cells covering many of the microparticles in the microtissues (Fig. 5A). Unlike nanoparticles that are typically internalized by cells, enabling intracellular delivery and signaling, microparticles largely remain extracellular and provide stable geometrical and structural cues within 3D microtissues. In this context, microparticles can also act as reservoirs for the controlled or sustained release of nanoparticles or soluble factors, helping mitigate potential cytotoxicity from nanoparticle exposure while enabling more controlled presentation of bioactive signals.

Fluorescence staining results revealed the formation of an organized F-actin network in microtissues containing composite microparticles, with the network conforming to the shape of the composite microparticles (Fig. 5B, Fig. S7). ALP and OPN immunofluorescent staining demonstrated higher intensity of the proteins in the microtissues containing composite microparticles, with those containing R-MC and S-MC exhibiting the highest ALP and OPN intensities, respectively. Quantification of the results normalized per number of cells in microtissues confirmed these trends, even though no significant differences were found among microtissues containing microparticles with various shapes. Nonetheless, higher ALP values for all assemblies containing microparticles and higher OPN values for assemblies containing L-MC and S-MC were observed as compared to the hMSC-only control (Fig. 5C and D). The highest OPN value was observed in microtissues containing S-MC microparticles, which might contribute from their combination of high surface area (over three times that of L-MC) and the established role of CaP in upregulating OPN in a 3D osteogenic environment [65]. ALP activity showed higher values in hybrid microtissues compared to the hMSC-only control. Notably, the composite microparticles with R-MC and S-MC shapes led to higher ALP activity than those with L-MC shape (Fig. 5E). The highest ALP activity in hMSCs induced by R-MC can be attributed to their higher aspect ratio compared to L-MC and S-MC, which may promote hMSC elongation across the microparticles. Notably, altering hMSC morphology has been previously reported to induce hMSC differentiation into the osteogenic lineage [66,67].

The calcium ion (Ca^2+^) content in the collected cell culture medium was measured throughout the microtissue culture period, and the cumulative Ca^2+^ release was calculated. Results showed a consistent Ca^2+^ release with a similar trend in microtissues containing composite microparticles. Although the L-MC group demonstrated the highest cumulative Ca^2+^ release by day 10, no significant differences were observed among the three groups at any time point, suggesting a consistent and progressive release profile (Fig. 5F). In summary, while all composite microparticles with different sizes and aspect ratios induced hMSC osteogenic differentiation, the S-MC induced the highest value for OPN intensity, and the R-MC induced the highest value for ALP expression, indicating the potential of these two parameters for promoting hMSC differentiation into osteogenic lineage. Nevertheless, it should be noted that fabricating particles with other geometrical parameters, such as curvatures (e.g., conical or spherical shapes), remains challenging with our current method. Future studies could explore alternative strategies, for instance, integrating 3D-printed molds with complex architectures into the existing fabrication workflow, to further investigate the influence of more diverse particle geometries on stem cell differentiation within 3D microtissues. Regarding the fabrication of particles with other sizes, photolithography enables the fabrication of molds at the nanometer scale. However, our manufacturing process requires the replacement of air within the mold, which imposes practical constraints; that is, as the target feature size decreases, the fabrication process becomes increasingly challenging with the composite biomaterial. At present, the minimum achievable composite microparticle size using this approach remains to be systematically determined.

### 3.4 Effect of composite microparticle shape on hMSC osteogenic differentiation in hybrid hMSC-composite microparticle microtissues

**Fig. 6:**
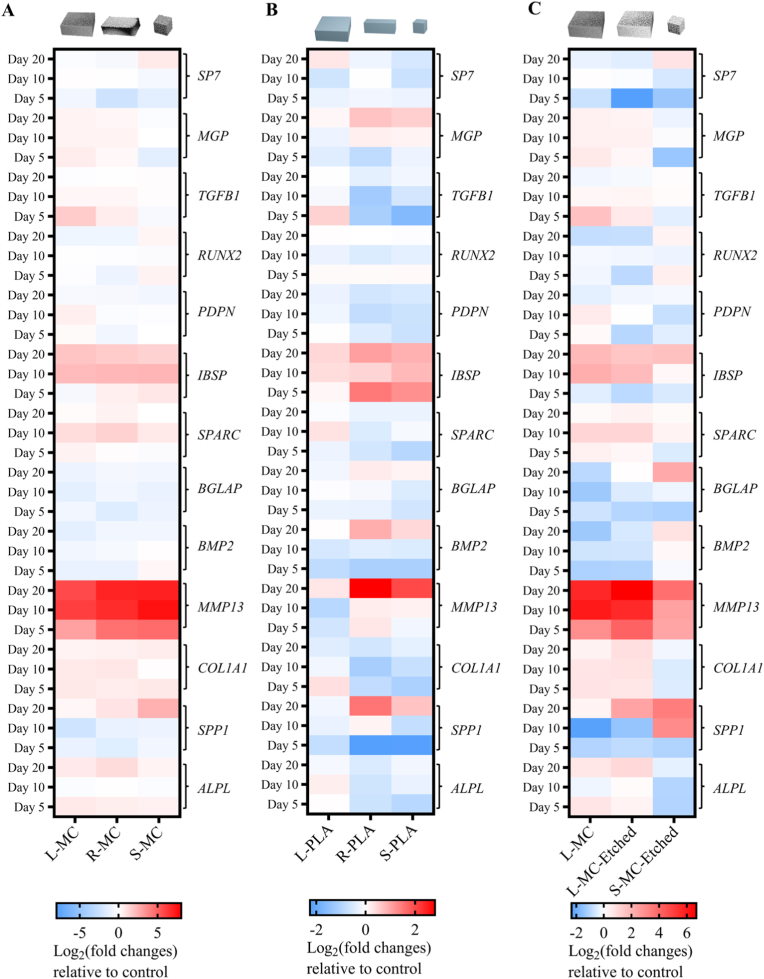
Effect of microparticle shape and chemistry on mRNA expression of osteogenic genes in hMSC microtissues. Heatmaps of mRNA expression of a panel of osteogenesis-related genes on days 5, 10, and 20 for microtissues containing (A) composite microparticles with a PLA/nHA ratio of 30/50 and different shapes, (B) PLA microparticles with different shapes, and (C) surface-etched composite microparticles with PLA/nHA ratio of 30/50 and different sizes. Fold changes are expressed relative to the mRNA levels measured in the cell-only control microtissues at the same time point.

**Fig. 7:**
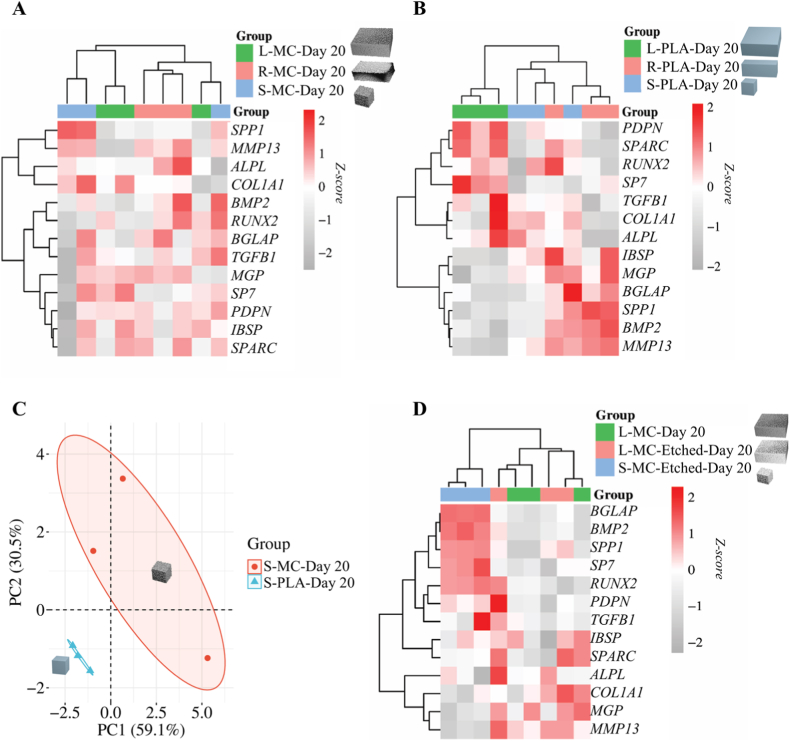
Cluster and PCA analyses of mRNA expression of osteogenic genes in hMSC microtissues. Cluster heatmap of gene expression on day 20 in hybrid microtissues containing (A) composite microparticles with a PLA/nHA ratio of 30/50 and different shapes and (B) PLA microparticles with different shapes. PCA plot of gene expression in hybrid microtissues containing L-MC, L-MC-Etched, and S-MC-Etched microparticles on day 20. (D) Cluster heatmap of gene expression on day 20 in hybrid microtissues containing surface-etched composite microparticles with PLA/nHA ratio of 30/50 and different shapes.

To determine whether microparticle shape affects the osteogenic differentiation of hMSCs, we profiled the expression of key osteogenic genes in microtissues containing three shapes (L-MC, R-MC, and S-MC) over 20 days (Fig. 6A). Our results revealed that compared to cell-only microtissue, all composite microparticles enhanced hMSC osteogenesis, with each shape preferentially promoting a distinct stage of gene expression. Specifically, the highest value of *ALPL* expression was seen in microtissue with R-MC on day 20, in line with our previous ALP intensity and activity detection on day 10, although no significant differences were found among various conditions. The highest value of *MMP13* was detected in microtissue containing S-MC on all time points, with significant improvement compared to L-MC on days 10 and 20, plausibly indicating that matrix remodeling [68] and osteogenic differentiation [69] were promoted by S-MC groups. Notably, the mRNA expression of *SPP1*, which encodes OPN, was elevated in the R-MC (1.33-fold) and S-MC (4.50-fold) groups compared to the L-MC group (Fig. 6A, Fig. S10A) on day 20. This is in line with the earlier observations about higher OPN production in conditions with S-MC (Fig. 5D), suggesting bone remodeling and biomineralization, associated with OPN [70,71], is potentially promoted by S-MC microparticles. Upregulation of *SPP1* and *MMP13* in S-MC group was consistent with the production of OPN and MMP13 at protein level, which was detected with the multiplex protein assay (Fig. S8). In addition to OPN and MMP13, the production of RANKL and GAS6 proteins were also measured. Results demonstrated microtissues containing S-MC had significantly higher RANKL production compared to both cell-only microtissues and the L-MC group on day 10 (Fig. S8), possibly indicating S-MC contributed to bone remodeling and metabolism [72]. Microtissues demonstrated similar GAS6 production among all groups, suggesting there is no impaired cell viability induced by composite microparticle. To further identify the conclusive difference among three groups on the mRNA level, we performed clustering analysis of the gene expression results on day 20. All composite microparticles improved hMSC osteogenic differentiation to a certain extent, with upregulated genes in S-MC associated with bone matrix remodeling (*SPP1*, *MMP13*, and *COL1A1*), in R-MC associated with osteoblast differentiation (*ALPL*, *BMP2*, and *RUNX2*), and in L-MC associated with potential mineralization (*MGP*, *SP7* and *PDPN*). However, the lack of clear clustering among the three groups on day 20 (Fig. 7A) implied that the size and aspect ratio of composite microparticles do not cause a broad, concerted shift in gene expression, which can possibly be related to the strong effect of bioactive chemistry of composite microparticles. Therefore, we removed the nHA from microparticle composition to further study the size and aspect ratio effect on osteogenic gene expression.

In order to decouple the shape effect from the bioactive chemistry of nHA, we prepared PLA microparticles with the large cube, rectangular prism and small cube shapes (labeled as L-PLA, R-PLA, and S-PLA, respectively) using the described micromolding method and analyzed the relative mRNA expressions of the osteogenesis-related genes on days 5, 10, and 20 (Fig. 6B, Fig. S10B). *MMP13* and *BMP2* [73,74] demonstrated significantly higher expression in the R-PLA group on day 20 compared to L-PLA, suggesting the potential of R-PLA for inducing higher BMP2 at the protein level, which has been shown to lead to bone formation [73,74]. Furthermore, the highest expression of *SPP1*, *IBSP*, and *MGP* on day 20 was found in microtissues containing R-PLA. In the heatmap of cluster analysis (Fig. 7B), the osteogenic gene expression profile of the L-PLA group was distinct from those of the R-PLA and S-PLA groups. Specifically, improved expressions of *IBSP*, *MGP*, *BGLAP*, *SPP1*, *BMP2*, and *MMP13*, were primarily associated with the R-PLA group. These results indicated a pronounced aspect ratio-dependent influence on osteogenesis-related gene expression in the absence of nHA, with R-PLA microparticles eliciting the highest overall expression levels for many osteogenic genes. Meanwhile, PCA was conducted to assess the differences in gene expression profiles, revealing a clear separation in gene expression clusters between S-PLA and S-MC groups on day 20 (Fig. 7C). This indicates that the introduction of nHA into microparticles substantially alters the aspect ratio-dependent gene expression. However, it is important to note that the thickness of PLA microparticles is overall less than that of composite microparticles due to the previously described coffee-ring effect, and this may also be an additional contributing factor in the results obtained above.

It should be noted that the bioactive nHA component in the composite microparticles was dispersed in a polymeric matrix, which can obscure nHA and limit its direct exposure to cells to some extent. This can be alleviated by surface etching with an alkaline solution, such as NaOH, which was previously shown to increase the exposure of HA and hydrophilicity at the surface of a poly(lactic-co-glycolic acid)-HA composite [75]. Therefore, we etched the surfaces of L-MC and S-MC composite microparticles with a NaOH/ethanol solution (labeled as L-MC-Etched and S-MC-Etched) and co-aggregated them with hMSCs to form microtissues as was described earlier. A significant increase in the amount of Ca measured by EDS at the surface of the composite microparticles after approximately 3 min of etching was observed (Fig. S9). Notably, *SPARC* expression was upregulated in both L-MC and L-MC-Etched microparticles on day 10, suggesting that the composite microparticles with large cube shape may promote hMSC osteogenesis via SPARC-mediated procollagen processing and matrix mineralization facilitated by SPARC's calcium-binding domains [76]. Of interest, *SPP1* expression in S-MC-Etched was significantly higher on days 10 and 20, and on day 20, *BMP2* and *BGLAP* expression in S-MC-Etched was significantly higher than in L-MC and L-MC-Etched (Fig. 6C, Fig. S11). *BGLAP* encodes osteocalcin that regulates bone remodeling and energy metabolism, which demonstrated the highest expression in S-MC-Etched group. *MMP13* expression, on the other hand, exhibited the lowest expression in the same group. The observed suppression of other genes, especially *ALPL,* in the S-MC-Etched group might be attributed to promoter methylation, a mechanism known to influence osteoblast lineage determination [77]. The expression of *RUNX2*, which is a master transcription factor in osteogenesis at protein level [78], was slightly higher in microtissues with S-MC-Etched microparticles than in those with L-MC-Etched (day 5) and L-MC (day 20) microparticles. The heatmap of the gene expression cluster analysis revealed that while L-MC and L-MC-Etched groups clustered together, a distinct gene profile was observed for S-MC-Etched groups on day 20 (Fig. 7D). These results suggested that compared to other microparticle groups, surface-etched S-MC could effectively enhance hMSC differentiation into a late osteogenic lineage, evidenced by highly expressed osteogenic genes found in mature osteoblasts and osteocytes, such as *MMP13*, *SPP1* and *BGLAP*.

Overall, these results underscore that both the shape and chemistry of microparticles serve as key design parameters in driving the osteogenic differentiation of hMSCs in hybrid microtissues, with their time-dependent effects on mRNA expression of osteogenic genes summarized in Fig. 9A. Specifically, compared to cell-only microtissue at the same day, in the absence of nHA, rectangular prism PLA microparticles (i.e., R-PLA) significantly enhanced osteogenic differentiation, primarily through the upregulation of *IBSP, SPP1* and *MMP13,* while large cube PLA (i.e., L-PLA) did not effectively improve any osteogenic gene expression. The differences in gene expression here are led by the anisotropic shape of the microparticles, a property that was previously shown to influence cell invasion in a granular particle system [13]. These results demonstrated that biophysical cues, such as microparticle size and aspect ratio, can serve as design criteria to direct hMSC differentiation without using costly growth factors, in agreement with earlier studies showing that microparticle topography drives and modulates osteogenesis [15]. Furthermore, surface etching amplified osteogenic effects in S-MC group by exposing nHA at the surface of the microparticles. Surface-etched S-MC microparticles may have favored late osteogenesis stage and matrix mineralization, as they induced *BGLAP* overexpression. For future directions, further surface modification of nHA, such as tailoring the hydration layer and modulating protein adsorption [79], or immobilizing nHA with other polymers [80], or combining with other functional nanoparticles [81], represents a promising strategy to enhance bone regeneration. These approaches may also confer additional benefits, including antibacterial properties and immunomodulatory effects.

**Fig. 8:**
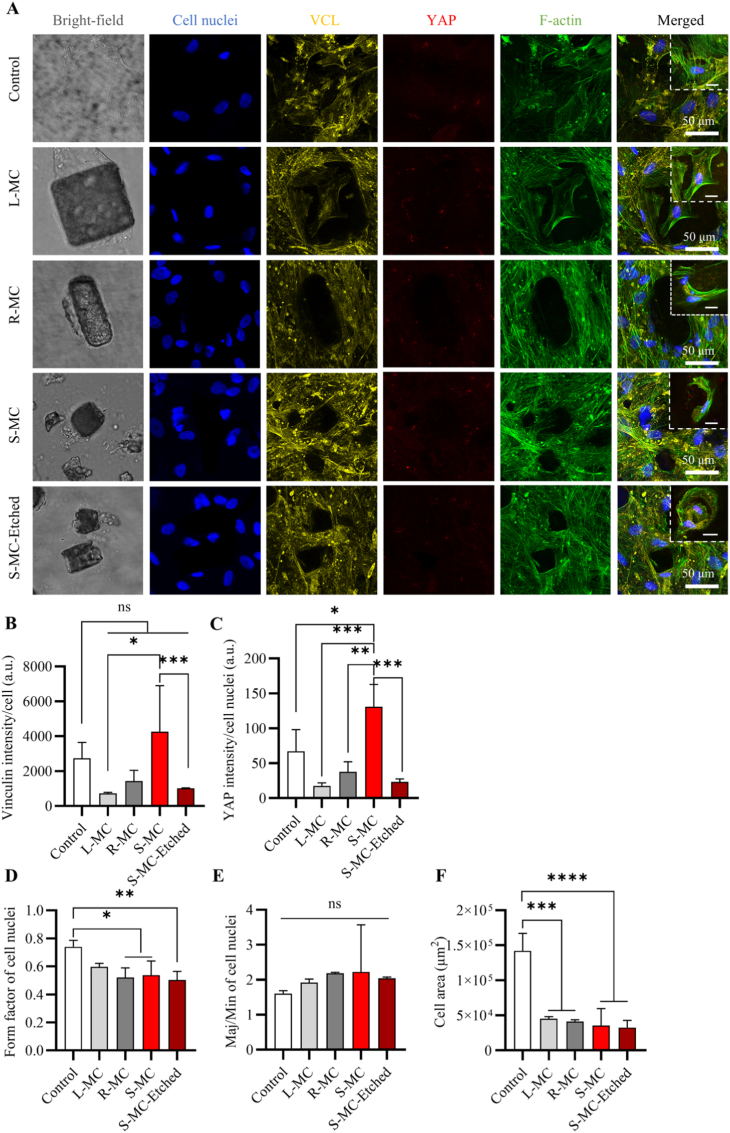
Characterization of hMSC culture on composite microparticles with PLA/nHA of 30/50 and different shapes in 2.5D. (A) Maximum intensity projections of confocal microscope images of cell cultured on microparticles in OM on day 10. Cell nuclei, VCL, YAP, and cytoskeletal F-actin are visualized in blue, yellow, red, and green, respectively. Brightfield images were used to visualize the microparticles. Scale bars apply to the images in the same row. Scale bars in the insets indicate 20 μm. Quantification of (B) VCL per cell and (C) YAP production per cell nuclei. Quantification of (D) Form factor and (E) Major/minor axis ratio (Maj/Min) of cell nuclei, and (F) cell area in each group. Data in B, C, D, E, and F were analyzed using a one-way ANOVA followed by Tukey's HSD post-hoc test (^ns^*p* > 0.05, ∗*p* < 0.05, ∗∗*p* < 0.01, ∗∗∗*p* < 0.001 and ∗∗∗∗*p* < 0.0001). ‘ns’ represents non-significant difference. (For interpretation of the references to colour in this figure legend, the reader is referred to the Web version of this article.)

**Fig. 9:**
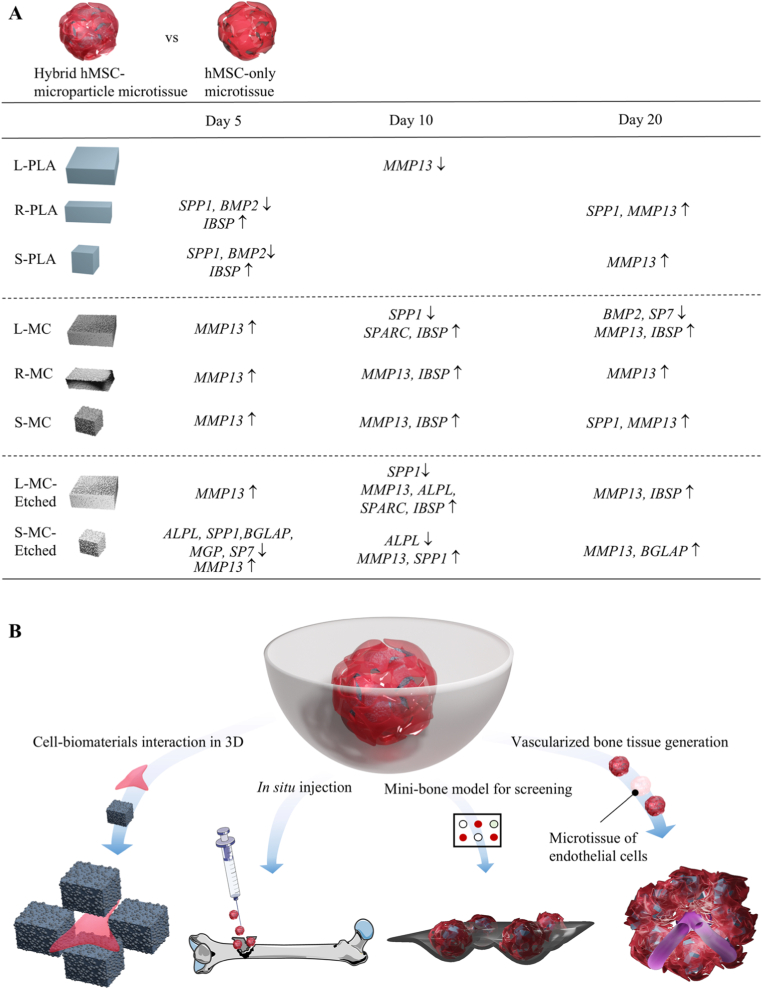
Summary of the effects of microparticle shape and chemistry on hMSC osteogenic differentiation, in hybrid microtissues and potential future directions. (A) A summary of gene expression in hMSC microtissues in response to microparticles with different chemistries and shapes, as compared to cell-only control microtissues at the same time point. Up- and downregulations of the genes are marked by “↑ -” and “↓”, respectively. Significance thresholds of |log_2_(fold change)| > 1 and *p* < 0.05 compared to cell-only control microtissues have been implemented. (B) Potential future direction for using shape-defined composite microparticles and the hybrid microtissues integrating them.

### 3.5 Composite microparticles influence hMSC mechanotransduction and nuclear morphology in 2.5D culture

To further explore the effect of microparticle size and aspect ratio on hMSC morphology and mechanotransduction, a 2.5D cell culture on microparticles was conducted. Immunostaining result revealed that on day 10, YAP was found in cell nuclei in all groups (Fig. 8A). Quantitative analysis showed that both YAP and VCL fluorescence intensities were significantly higher in the S-MC group compared to the L-MC group on day 10 (Fig. 8B and C), suggesting that enhanced mechanotransductive signaling may contribute to the elevated osteogenic markers observed in S-MC. However, YAP and VCL intensities in microtissues containing S-MC-Etched were markedly lower than those in the S-MC group. Although our current data does not permit a definitive explanation, we propose that YAP and VCL may be activated earlier in S-MC-Etched-containing microtissues, consistent with their earlier onset of osteogenic markers such as SPP1 expression, or YAP signaling may become uncoupled from osteogenic differentiation in response to altered surface chemistry. Indeed, previous studies have reported that YAP expression can be independent of certain scaffold parameters, such as matrix stiffness [82]. The relationship between YAP signaling and cell differentiation, particularly in the context of particle shape and surface chemistry, warrants further investigation.

The cell morphology analysis results showed a decrease in nuclear form factor in hMSCs cultured on microparticles, particularly R-MC, S-MC, and S-MC-Etched, compared to the flat control (Fig. 8D), suggesting that the nuclei became more elongated and irregular, deviating from a smooth, circular morphology, possibly indicating an increased cytoskeletal tension and actomyosin contractility. The unchanged major/minor axis ratio (Fig. 8E) suggests that this deformation is not uniformly oriented along a single axis. Instead, the increased irregularity likely results from multi-directional or local compressive forces exerted by the cytoskeleton as cells attach and conform to the microparticles. The consistent reduction in cell area (Fig. 8F) across all particle shapes, relative to flat controls, indicated that microparticles physically constrain cell spreading.

Taken together, these findings suggest that while all microparticle shapes impose physical constraints that limit overall cell spreading, the particle size and aspect ratio both contribute to distinct nuclear deformation and compression, particularly from multiple directions. Previous studies have shown that hMSCs with greater actomyosin contractility [21] or elongated morphology [83] in response to geometric cues preferentially undergo osteogenic differentiation. Our results evidenced our hypothesis that by tailoring microparticle shape, such as aspect ratio, size, and other complex features, at a cell-scale level, we can tune cell contractility and aspect ratio, and by integrating bioactive materials like nHA, we can ultimately modulate osteogenic commitment.

Inspired by the hybrid microtissue system developed in this work, several promising future directions are possible. For instance, our platform offers a robust approach for investigating hMSC-microparticle interactions in a physiologically relevant 3D microenvironment (Fig. 9B). The designed microparticles can be tailored to stimulate osteogenesis without the need for exogenous growth factors, and hence, show promise to be used as injectable bone fillers. Moreover, this platform can be adapted to generate bone-associated disease models for drug screening, either in a high-throughput format by integration in microwell arrays [84], or in a more physiologically relevant format in combination with organ-on-chip microfluidic devices [85]. Additionally, leveraging microtissues as modular building blocks for bioprinting offers a powerful strategy to engineer complex, vascularized bone tissues, which is supported by recent advances where microtissues and hydrogel have been successfully employed as bioinks to generate centimeter-scale bone constructs with early vascular networks [86].

## 4 Conclusions and perspective

This study investigated the roles of shape and surface chemistry of microparticles in driving the osteogenic differentiation of hMSCs within 3D microtissues. The micromolding fabrication method described here allowed precise replication of microparticles with varied geometries and compositions, specifically PLA and PLA-nHA composites with various PLA/nHA contents. All types of microparticles successfully co-aggregated with hMSCs, forming 3D microtissues. The integration of composite microparticles in the microtissues significantly enhanced the ALP activity of the cells as compared to cell-only control microtissues, indicating an early osteogenic commitment. Notably, ALP activity also varied with microparticle shape, with rectangular prism and small cube composite microparticles inducing higher ALP activity than large cube microparticles, despite having the same composition. Importantly, both microparticle shape and composition significantly influenced osteogenic gene expression in hMSC microtissues. In the absence of nHA, PLA microparticles with a higher aspect ratio induced the highest osteogenic gene expression, highlighting the intrinsic ability of physical cues to direct cell fate. The introduction of bioactive nHA and its subsequent surface exposure via etching made the small cube microparticles the most effective group for enhancing the expression of *BGLAP*, a late osteogenic marker, plausibly shifting the cellular response towards late-stage maturation and matrix mineralization. Mechanotransduction markers of hMSC were also found to be upregulated with microparticles in smaller sizes. Microparticle shape, especially size and aspect ratio, therefore, can play a central role in driving osteogenic differentiation of hMSCs in 3D microtissues, and this effect can be synergistically altered and enhanced by incorporating bioactive chemistries such as nHA in microparticles. These insights underscore the potential of shape- and chemistry-tuned microparticles as designer instructive biomaterials for bone regeneration without the need for using growth factors. Looking forward, one promising avenue for optimizing osteogenic outcomes would be to systematically explore mixtures of microparticles with varying sizes, aspect ratios, and surface areas. However, while our study provides compelling *in vitro* evidence, the therapeutic efficacy of the shape-defined microparticles awaits validation, for example, in critical-sized bone defect models. In future work, training a computational model based on a broader set of experimental parameters may help elucidate the efficacy of cell–microparticle surface contact and guide the optimization of microparticle shape for enhanced osteogenic outcomes.

## CRediT authorship contribution statement

**Ke Song:** Conceptualization, Formal analysis, Funding acquisition, Investigation, Methodology, Validation, Visualization, Writing – original draft, Writing – review & editing. **Maryam Parvizifard:** Formal analysis, Investigation, Methodology, Visualization, Writing – review & editing. **David Barata:** Methodology, Writing – review & editing. **Jiaping Li:** Methodology, Writing – review & editing. **Roman Truckenmüller:** Conceptualization, Funding acquisition, Methodology, Project administration, Supervision, Writing – review & editing. **Pamela Habibović:** Conceptualization, Funding acquisition, Supervision, Writing – review & editing. **Zeinab Niloofar Tahmasebi Birgani:** Conceptualization, Funding acquisition, Methodology, Project administration, Supervision, Writing – review & editing.

## Declaration of competing interest

The authors declare the following financial interests/personal relationships which may be considered as potential competing interests: Ke Song reports financial support was provided by China Scholarship Council (CSC) from the Ministry of Education of P.R. China. Zeinab Niloofar Tahmasebi Birgani reports financial support was provided by Dutch Research Council. Zeinab Niloofar Tahmasebi Birgani reports financial support was provided by Maastricht University and Academic Hospital Maastricht. Pamela Habibović reports financial support was provided by Dutch Research Council. Pamela Habibović reports financial support was provided by European Union Interreg Vlaanderen-Nederland. Pamela Habibović reports financial support was provided by Dutch Province of Limburg. Roman Truckenmüller reports a relationship with 300MICRONS that includes: board membership and equity or stocks. If there are other authors, they declare that they have no known competing financial interests or personal relationships that could have appeared to influence the work reported in this paper.

## Data Availability

Data will be made available on request.
