# Supplementary material for: Shape-defined poly(lactic acid)-nanohydroxyapatite composite microparticles modulate osteogenic differentiation in 3D microtissues of human mesenchymal stromal cells
Source: Mater Today Bio. 2026 Mar 26;38:103075. doi: 10.1016/j.mtbio.2026.103075 (PMC13087642; doi:10.1016/j.mtbio.2026.103075)
Supplement: Multimedia component 1 [file mmc1.docx]

**Supplementary data**

### **Shape-defined poly(lactic acid)-nanohydroxyapatite composite microparticles modulate osteogenic differentiation in 3D microtissues of human mesenchymal stromal cells**

Ke Song, Maryam Parvizifard, David Barata, Jiaping Li, Roman Truckenmüller, Pamela Habibović, Zeinab Niloofar Tahmasebi Birgani^*^

*MERLN Institute for Technology-Inspired Regenerative Medicine, Maastricht University, P.O. Box 616, 6200 MD, Maastricht, The Netherlands*

*Corresponding author, Email: [z.tahmasebibirgani@maastrichtuniversity.nl](mailto:z.tahmasebibirgani@maastrichtuniversity.nl)


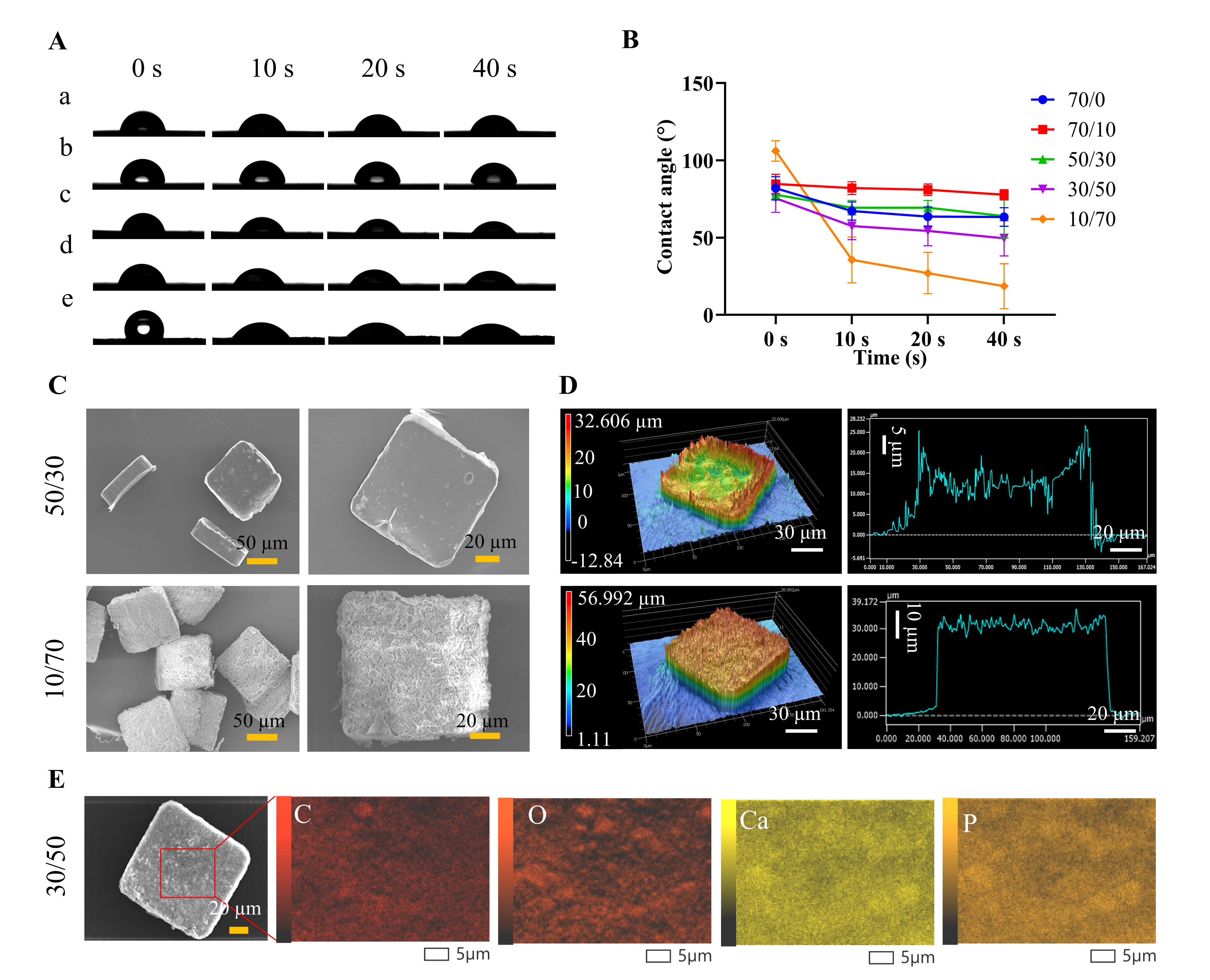


**Fig. S1.** (A) Representative images of a water droplet on (a) poly(lactic acid) (PLA) and composite films with PLA/nanohydroxyapatite (nHA) ratios of (b) 70/10, (c) 50/30, (d) 30/50, and (e) 10/70, and (B) quantification of the contact angle values at 0, 10, 20, and 40 s, indicating the wettability of different samples. (C) Scanning electron microscopy (SEM) images, and (D) height maps and profiles of composite microparticles with PLA/nHA of 50/30 (top) and 10/70 (bottom). (E) SEM image of composite microparticles with PLA/nHA of 30/50, and energy-dispersive X-ray spectroscopy (EDS) elemental maps of carbon (C), oxygen (O), calcium (Ca), and phosphorus (P) corresponding to an area selected on the surface of the microparticle.


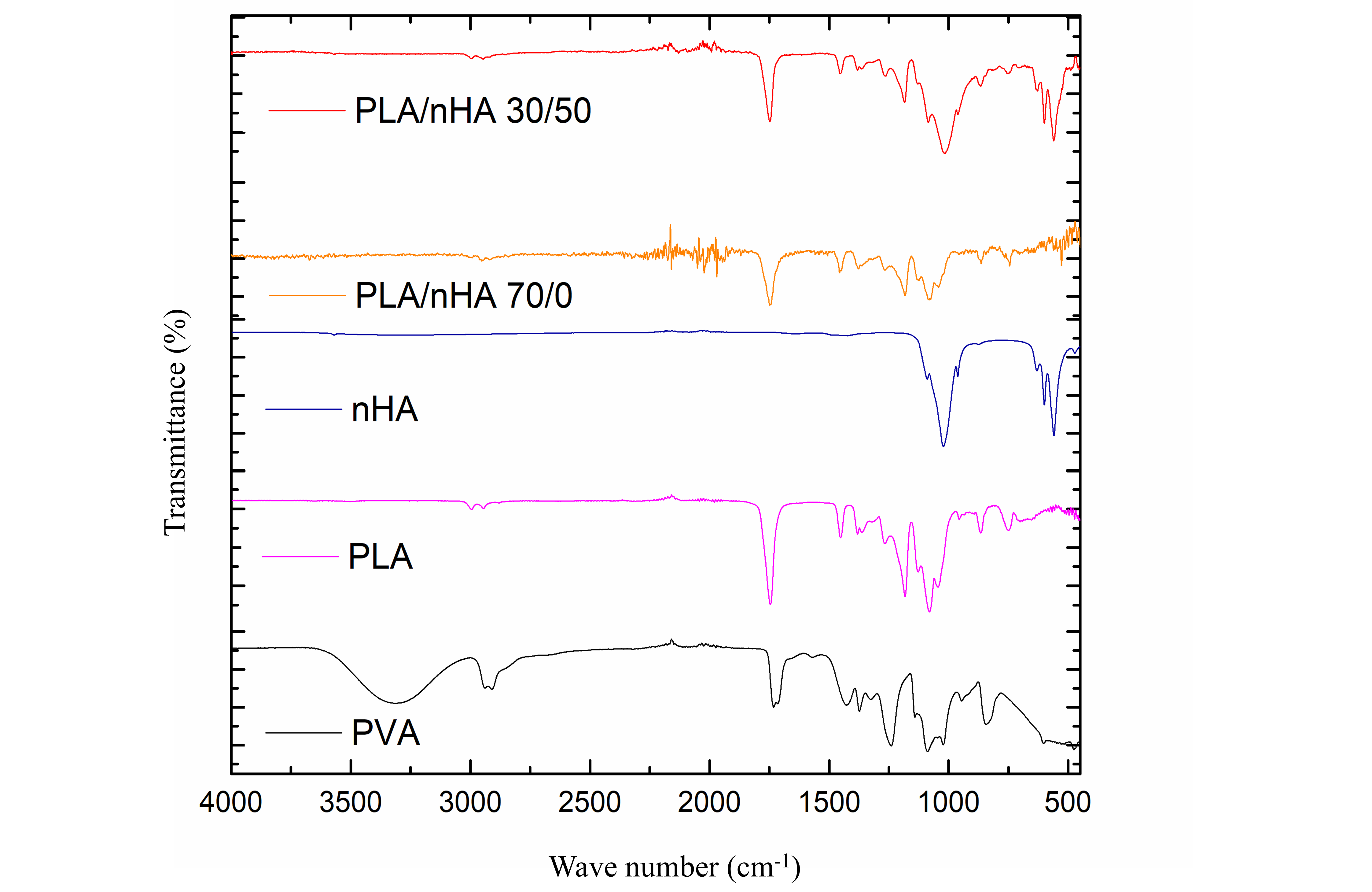


**Fig. S2.** Fourier transform infrared spectroscopy (FTIR) spectra of poly(vinyl alcohol) (PVA), PLA, nHA, PLA microparticles (PLA/nHA of 70/0), and composite microparticles with PLA/nHA of 30/50.


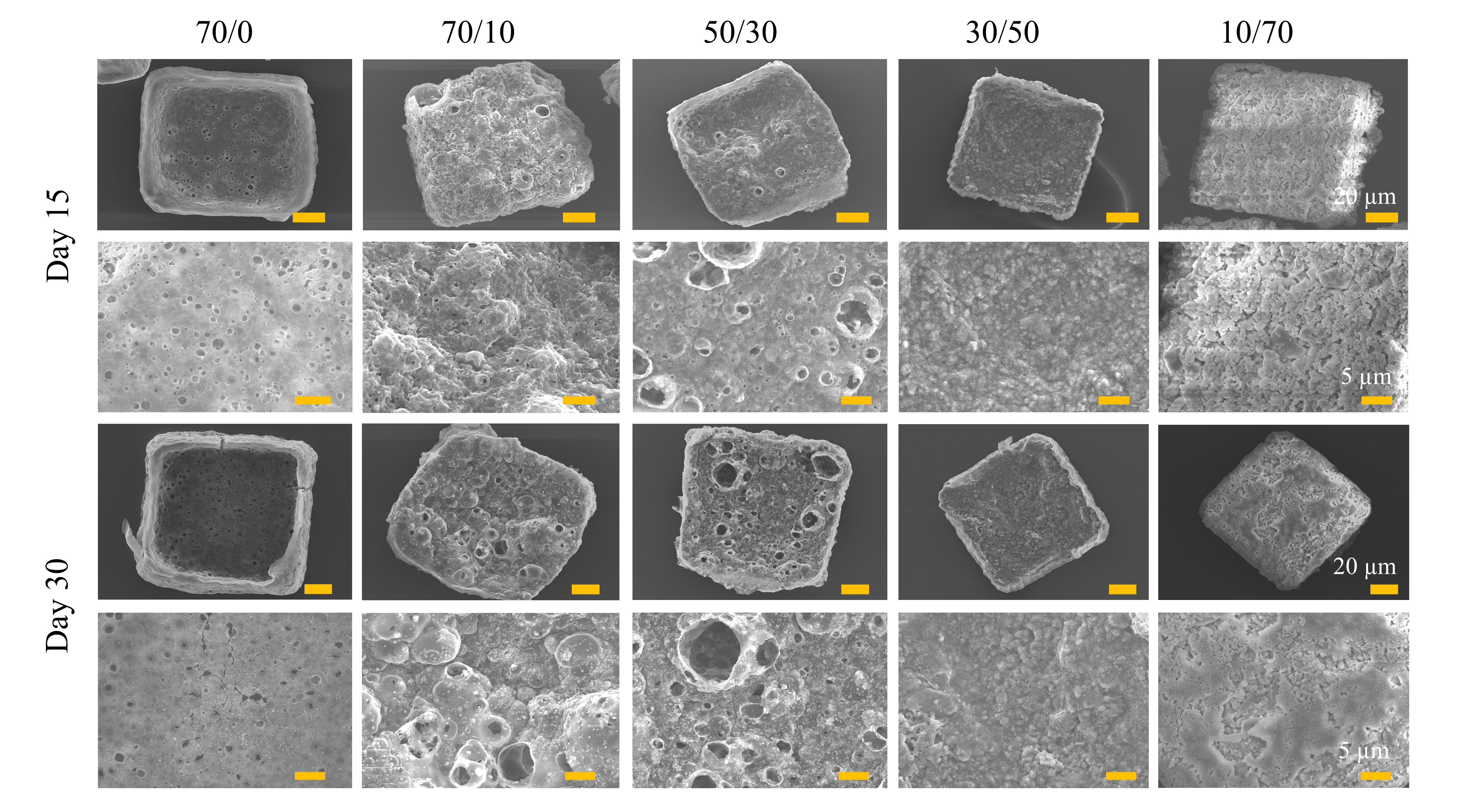
**Fig. S3.** SEM images of PLA microparticles (PLA/nHA of 70/0) and composite microparticles with PLA/nHA of 70/10, 50/30, 30/50, and 10/70 after incubation in cell culture medium for 15 and 30 days. Indications above the scale bars apply to the images in the same row.

**Fig. S4.** Surface roughness (Sa) of non-sterilized and UV-sterilized composite microparticles with PLA/nHA of 30/50. ‘ns’ represents non-significant difference.


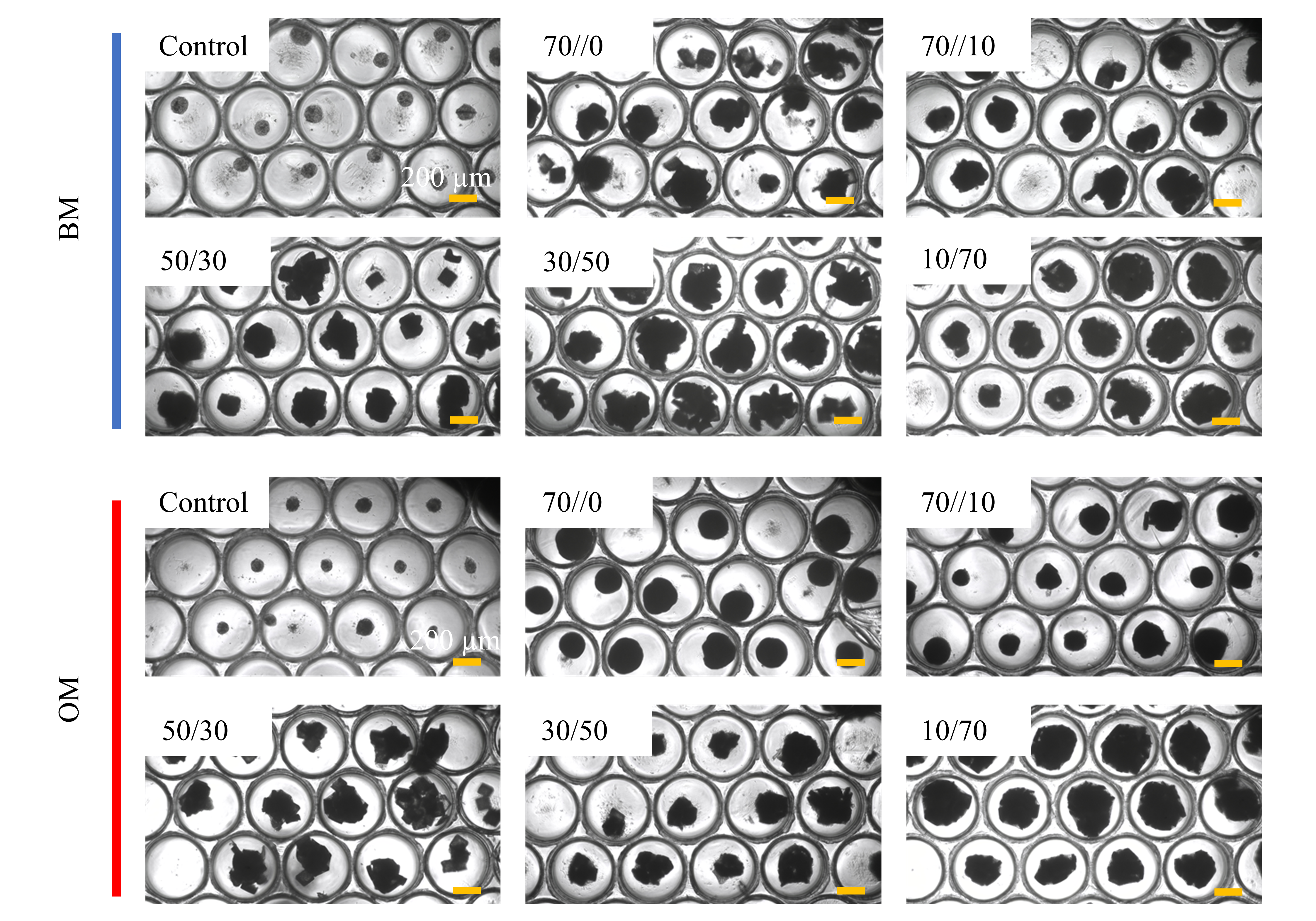
**Fig. S5.** Bright-field images of control hMSC-only and hybrid hMSC microtissues containing PLA microparticles (70/0) and composite microparticles with PLA/nHA of 70/10, 50/30, 30/50, and 10/70 in basic (BM) or osteogenic (OM) medium on day 10. Indications above the scale bars apply to all images.


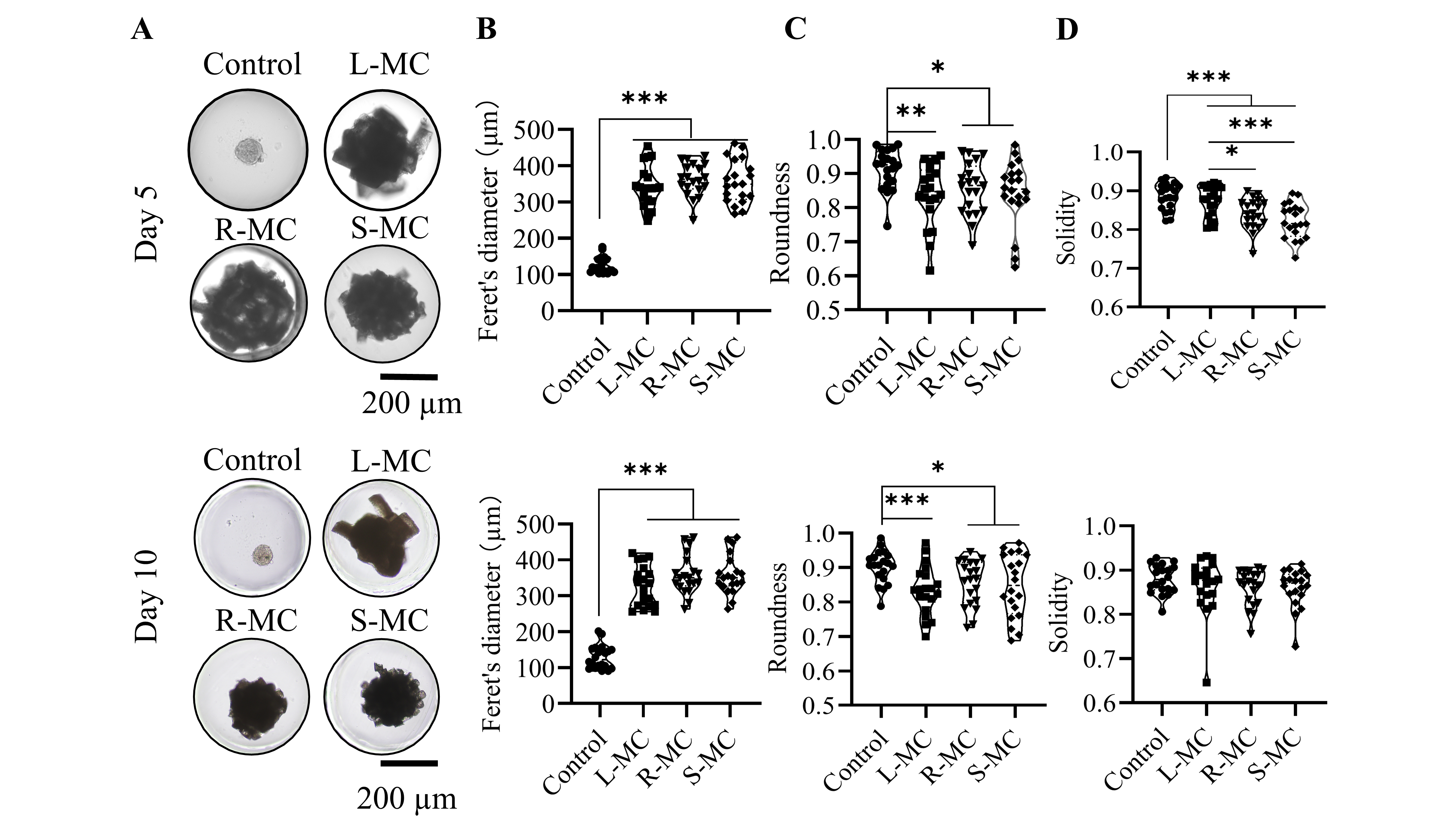


**Fig. S6.** (A) Bright-field images of control hMSC-only and hybrid hMSC microtissues containing composite microparticles with PLA/nHA of 30/50, and L-MC, R-MC, and S-MC shapes in osteogenic medium on days 5 (top) and 10 (bottom). The scale bar applies to all images. (B) Feret’s diameter, (C) Roundness, and (D) Solidity of the microtissues on days 5 (top) and 10 (bottom). Data in B, C, and D were analyzed using a one-way ANOVA followed by a Tukey’s HSD post-hoc test (* *p*<0.05, ** *p*<0.01, and *** *p*<0.001).


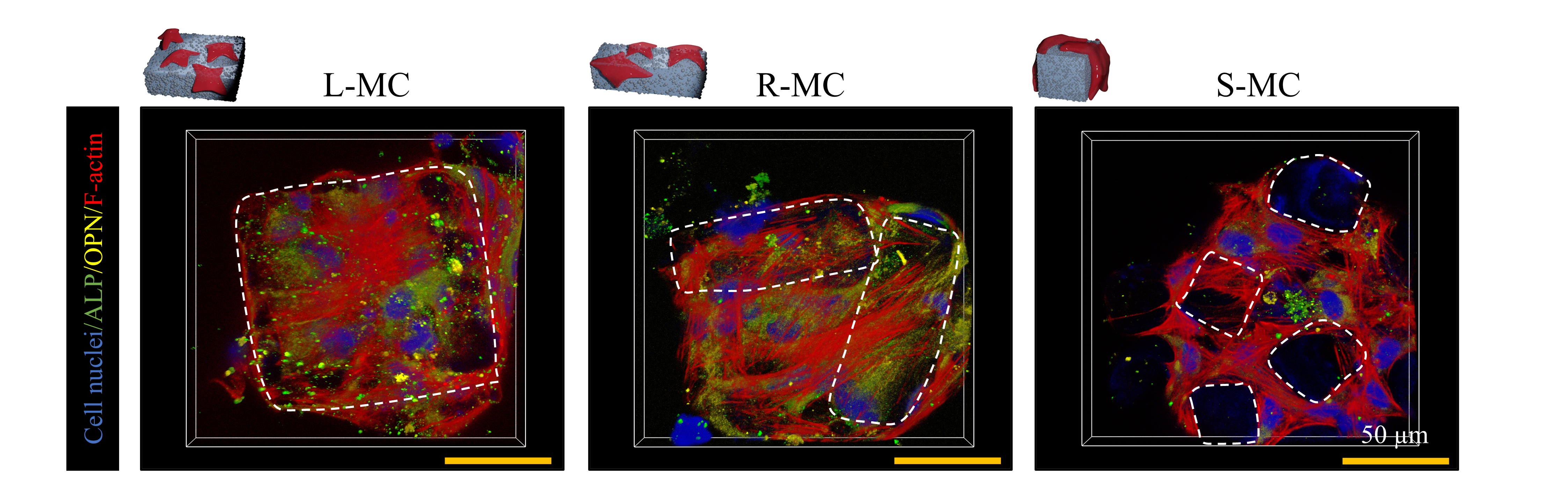


**Fig. S7.** Maximum intensity projections of confocal fluorescence microscopy images of hybrid microtissues containing microcomposites with PLA/nHA ratio of 30/50, and L-MC, R-MC, and S-MC shapes on day 10. Cell nuclei, alkaline phosphatase (ALP), osteopontin (OPN), and cytoskeletal F-actin are visualized in blue, green, yellow, and red, respectively. White dashed lines represent the periphery of composite microparticles. Indications above the scale bar apply to all images. Z-stack =20 µm.


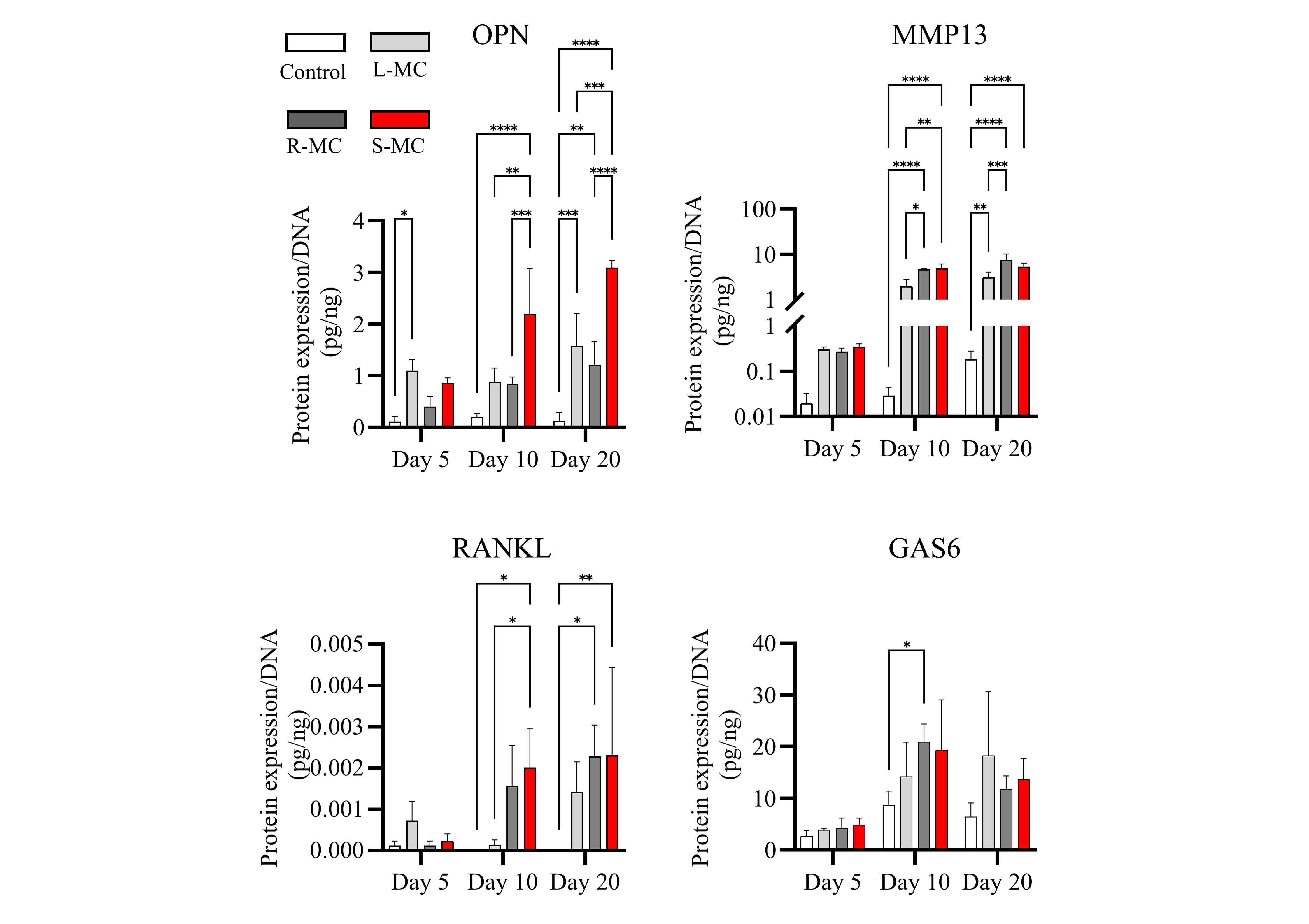


**Fig. S8.** Production of OPN, MMP13, RANKL, and GAS6 proteins on days 5, 10, and 20 in hybrid microtissues containing L-MC, R-MC, and S-MC with PLA/nHA of 30/50.

**Fig. S9.** Calcium mass ratios on either side of L-MC and L-MC-Etched composite microparticles, quantified by EDS.


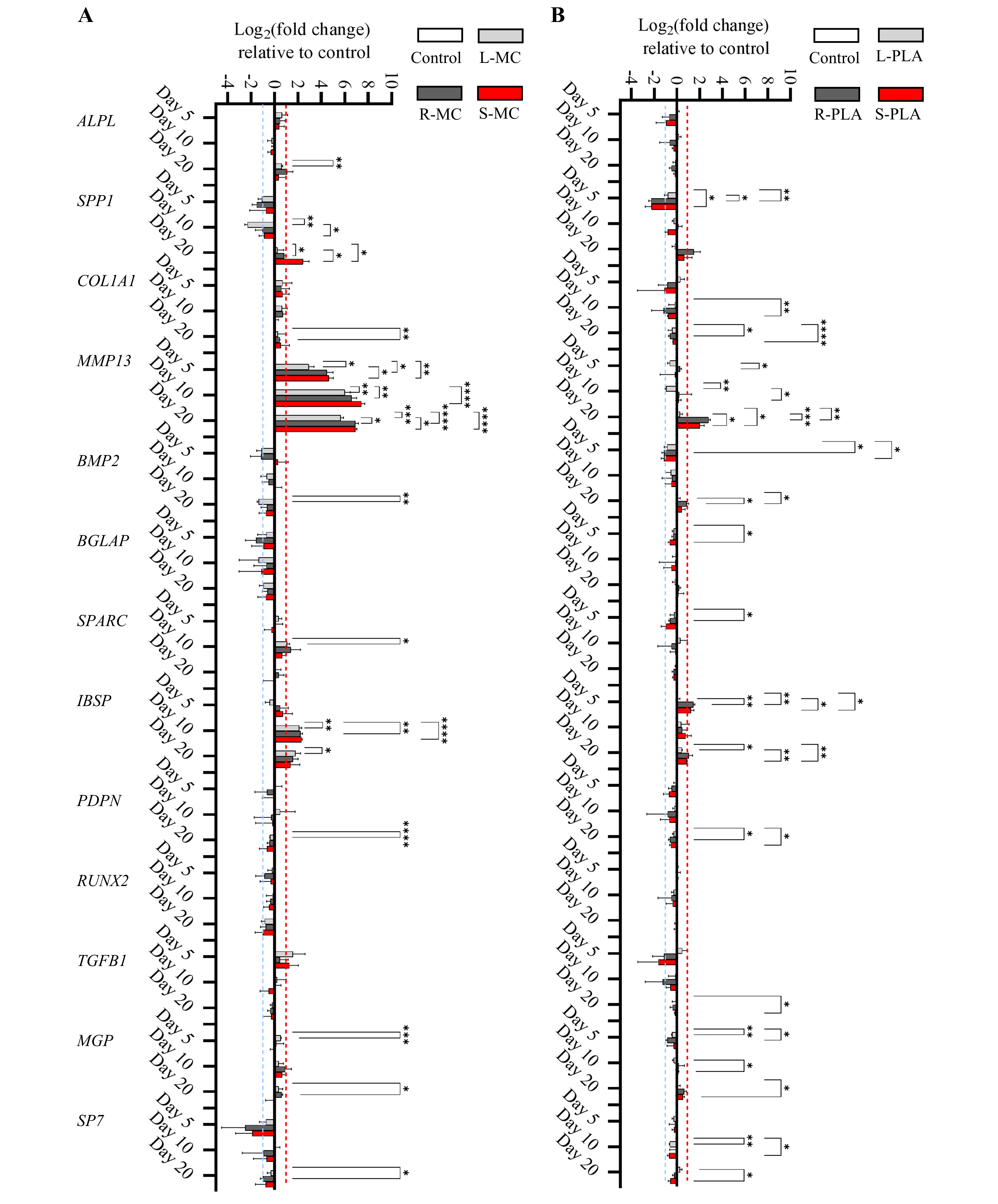


**Fig. S10.** Bar graph showing expression of a panel of osteogenesis-related genes at days 5, 10, and 20 for microtissues containing (A) composite microparticles with a PLA/nHA ratio of 30/50 and different shapes, (B) PLA microparticles with different shapes. Fold changes are expressed relative to the mRNA levels measured in the cell-only control microtissues at the same time point. Dash lines (in red and blue) represent significance thresholds of |log2(fold change)| > 1 and *p* < 0.05 compared to cell-only control microtissues. Data were analyzed using a two-way ANOVA followed by a Tukey’s HSD post-hoc test (* *p*<0.05, ** *p*<0.01, *** *p*<0.001 and **** *p*<0.0001).


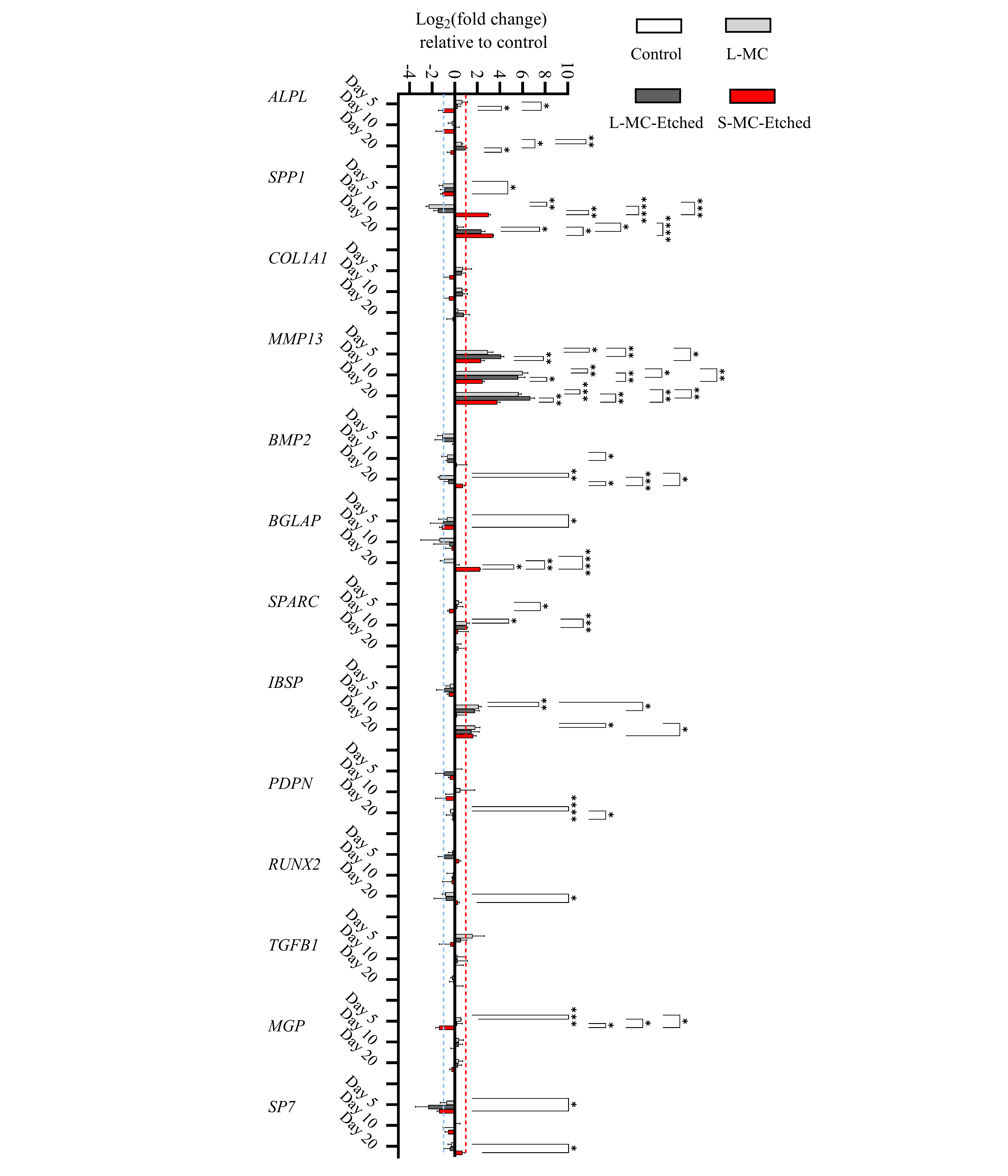


**Fig.S11.** Bar graph showing expression of a panel of osteogenesis-related genes at days 5, 10, and 20 for microtissues containing surface-etched composite microparticles with PLA/nHA ratio of 30/50 and different shapes. Fold changes are expressed relative to the mRNA levels measured in the cell-only control microtissues at the same time point. Dash lines (in red and blue) represent significance thresholds of |log2(fold change)| > 1 compared to cell-only control microtissues. Data were analyzed using a two-way ANOVA followed by a Tukey’s HSD post-hoc test (* *p*<0.05, ** *p*<0.01, *** *p*<0.001 and **** *p*<0.0001).

**Table S1.** Primer sequences used for determining the mRNA expression of osteogenic markers using RT-qPCR. For each marker, forward and reverse primer sequences are marked by F and R, respectively, and the annealing temperature is indicated.

| Gene symbol | Gene name | Sequence (5’-3’) | Annealing temperature (˚C) |
| --- | --- | --- | --- |
| *ALPL* | Alkaline phosphatase (tissue non-specific) | F: ACAAGCACTCCCACTTCATC  R: TTCAGCTCGTACTGCATGTC | 60 |
| *SPP1* | Secreted phosphoprotein 1 | F: GAAGTTTCGCAGACCTGACAT  R: GTATGCACCATTCAACTCCTCG | 60 |
| *BMP2* | Bone morphogenetic protein 2 | F: ACTACCAGAAACGAGTGGGAA  R: GCATCTGTTCTCGGAAAACCT | 60 |
| *COL1A1* | Collagen type I alpha 1 chain | F: GAGGGCCAAGACGAAGACATC  R: CAGATCACGTCATCGCACAAC | 62 |
| *MMP13* | Matrix metallopeptidase 13 | F: CCAGACTTCACGATGGCATTG  R: GGCATCTCCTCCATAATTTGGC | 62 |
| *IBSP* | Integrin binding sialoprotein | F: CCCCACCTTTTGGGAAAACCA  R: TCCCCGTTCTCACTTTCATAGAT | 62 |
| *MGP* | Matrix Gla protein | F: TCCGAGAACGCTCTAAGCCT  R: GCAAAGTCTGTAGTCATCACAGG | 60 |
| *RUNX2* | Runt-related transcription factor 2 | F: TGGTTACTGTCATGGCGGGTA  R: TCTCAGATCGTTGAACCTTGCTA | 62 |
| *TGFB1* | Transforming growth factor beta 1 | F: GGCCAGATCCTGTCCAAGC  R: GTGGGTTTCCACCATTAGCAC | 62 |
| *PDPN* | Podoplanin | F: AGACACGGGAGAATACTTTTGC  R: AGTTCCTCGGCATCATTAGGG | 62 |
| *BGLAP* | Bone gamma-carboxyglutamate protein | F: TGAGAGCCCTCACACTCCTC  R: CGCCTGGGTCTCTTCACTAC | 60 |
| *SPARC* | Secreted protein acidic and rich in cysteine | F: TCGGCATCAAGCAGAAGGATA  R: CCAGGCAGAACAACAAACCAT | 62 |
| *SP7* | Sp7 transcription factor | F: CCTCTGCGGGACTCAACAAC  R: AGCCCATTAGTGCTTGTAAAGG | 60 |
| *TBP* | TATA-box binding protein | F: CCACTCACAGACTCTCACAAC  R: CTGCGGTACAATCCCAGAACT | 62 |
